# A 3-step static mechanistic modeling approach to predicting transporter- and enzyme-mediated interaction signals of direct oral anticoagulants

**DOI:** 10.1016/j.dmd.2026.100360

**Published:** 2026-07-06

**Authors:** Mostafa ben Ahmed, Anne M. Filppula, Aleksi Tornio

**Affiliations:** 1 Integrative Physiology and Pharmacology, Institute of Biomedicine, University of Turku, Turku, Finland; 2 Unit of Clinical Pharmacology, Turku University Hospital, Turku, Finland; 3 Pharmaceutical Sciences Laboratory, Department of Natural and Health Sciences, Faculty of Science and Engineering, Åbo Akademi University, Turku, Finland

**Keywords:** Pharmacokinetics, Drug interactions, Direct oral anticoagulants, In silico models, Efflux transporters, Metabolizing enzymes

## Abstract

Direct oral anticoagulants (DOACs) are susceptible to drug-drug interactions involving both transport and metabolism. The relative contribution of intestinal transporters in DOAC pharmacokinetics, however, is uncharacterized, hindering the prediction of their drug interactions. In this study, we examined potential interactions of select precipitants with apixaban, dabigatran, edoxaban, and rivaroxaban using a 3-step static mechanistic combinatorial modeling approach. In this approach, we (1) calculated apparent estimates of the fractions of efflux transport (*f*_e_) of the 4 DOACs by the intestinal breast cancer resistance protein and intestinal permeability glycoprotein (P-gp), (2) validated our estimates, and (3) used them to simulate unstudied drug interactions with the anticoagulants. We estimated P-gp and breast cancer resistance protein transport to play a similar role in the interactions of apixaban and rivaroxaban (*f*_e_P-gp_ = 0.21, *f*_e_breast cancer resistance protein_ = 0.13). Additionally, the role of P-gp was prominent in the interactions affecting dabigatran etexilate (*f*_e_P-gp_ = 0.61) and less so in those affecting edoxaban (*f*_e_P-gp_ = 0.38). Using our apparent estimates, we predicted 144 interaction signals of 47 drugs with the 4 anticoagulants. All signals showed potential change >25% in the area under the curve of DOACs, warranting further investigation. Using our combined model, we were able to uncover new drug interaction signals, including previously unrecognized signals by kinase inhibitors.

**Significance Statement:**

Multiple enzymes and transporters affect direct oral anticoagulant pharmacokinetics. Drugs interacting with the same proteins may increase the levels of direct oral anticoagulants, leading to bleeding complications. Current methods predicting such interactions focus only on individual enzymes or transporters, limiting the prediction of cases where both may be involved. In this study, we combined multiple models to capture the complexity of interactions affecting direct oral anticoagulants. Using our validated model, we predicted 144 drug-drug interaction signals with potential clinical significance.

## 1 Introduction

Direct oral anticoagulants (DOACs) are widely used for the treatment and prevention of thromboembolic diseases.^1^ In contrast to warfarin, their elimination is only partially dependent on cytochrome P450 metabolism. DOACs are additionally known substrates of the efflux transporters breast cancer resistance protein (BCRP) and permeability glycoprotein (P-gp).^2^, ^3^, ^4^, ^5^, ^6^, ^7^

Bleeding is the most clinically significant adverse effect of DOACs. It is a concentration-dependent effect, which may result from inhibiting DOAC metabolism and transport. This may be confirmed by the collective of in vitro and clinical evidence, which suggests a simultaneous inhibition of DOAC-metabolizing enzymes and efflux transporters in clinically significant interactions, which in turn increases the levels of DOACs.^3^, ^4^, ^5^, ^6^^,^^8^, ^9^, ^10^, ^11^, ^12^, ^13^, ^14^

Although the role of metabolizing enzymes in the pharmacokinetic drug interactions of DOACs is well characterized, the exact contribution of intestinal efflux transporters is not clear. Additionally, the common practice of evaluating single enzymes or transporters in static modeling studies further limits the capture of potential drug-DOAC interaction signals where multiple proteins may be involved.

In this work, we focus on predicting potential drug interactions involving apixaban, dabigatran, edoxaban, and rivaroxaban as objects of pharmacokinetic drug interactions. We use a systematic 3-step approach where we (1) establish the role of intestinal transporters in drug-DOAC interactions by estimating the fractions of the drugs transported by BCRP and P-gp using clinical observations. We then (2) validate our estimates using additional clinical drug interaction observations and (3) predict new drug interaction signals of DOACs as object drugs (an affected drug in a drug interaction) with select precipitants. In all 3 steps, we use a static mechanistic model which considers the cumulative effect of modulating all potentially involved intestinal transporters and intestinal and hepatic metabolizing enzymes.

## 2 Materials and methods

### 2.1 Data and software

In all 3 steps of this study, we used the Certara Drug Interaction Database (DIDB, Certara, accessed between September 2023 and June 2024) to collect clinical drug interaction observations, precipitant maximum plasma concentrations, enzyme and transporter reversible inhibition kinetics, and enzyme time-dependent and induction kinetics. Additional model variables were collected from various sources (Supplemental Tables 1–7). All analyses and calculations in this study were made in Microsoft Excel (Microsoft Corporation, version 2410 build 16.0.18129.20200).

### 2.2 Static mechanistic model

In all 3 steps of this study, we used a combinatorial model (eq. 1) combining the equation by Fahmi et al,^15^ which described the reversible inhibition, time-dependent inhibition, and induction of gut (_*g*_) and hepatic (_*h*_) metabolizing enzymes; and the equation validated by Zamek-Gliszczynski et al,^16^ to describe the reversible inhibition of efflux gut transporters. The inclusion of a transporter time-dependent or induction component was limited by the lack of a validated equation describing the same mechanisms.

Both component equations were adjusted to include multiple metabolizing enzymes and multiple transporters according to the work by Isoherranen et al.^17^ The Fahmi equation was further adapted to include the effect of reversible and time-dependent metabolite inhibitions as previously demonstrated by Paludetto et al.^18^

(1) $$AUCR=\left[\frac{1}{\sum_{a=1}^{n}{\left(\frac{1}{1+\frac{{\left[I\right]}_{g}}{K_{i}}}\times \frac{k_{\deg,g}}{k_{\deg,g}+\frac{{\left[I\right]}_{g}\times k_{inact}}{{\left[I\right]}_{g}+K_{I}}}\times \left(1+\frac{d\times E_{\max}\times {\left[I\right]}_{g}}{{\left[I\right]}_{g}+{EC}_{50}}\right)\times \left(1-F_{g}\right)\right)}_{a}+{\left(F_{g}\right)}_{a}}\right]\times \left[\frac{1}{\sum_{a=1}^{n}{\left(\frac{1}{1+\sum_{b=1}^{m}{\left(\frac{{\left[I\right]}_{h}}{K_{i}}\right)}_{b}}\times \frac{k_{\deg,h}}{k_{\deg,h}+\sum_{c=1}^{x}{\left(\frac{{\left[I\right]}_{h}\times k_{inact}}{{\left[I\right]}_{h}+K_{I}}\right)}_{c}}\times \left(1+\frac{d\times E_{\max}\times {\left[I\right]}_{h}}{{\left[I\right]}_{h}+{EC}_{50}}\right)\times f_{m}\right)}_{a}+\left(1-\sum_{a=1}^{n}{\left(f_{m}\right)}_{a}\right)}\right]\times \left[\frac{1}{\sum_{t=1}^{p}{\left(\frac{1}{1+\frac{{\left[I\right]}_{g}}{K_{i}}}\times {\;f}_{e}\right)}_{t}+\left(1-\sum_{t=1}^{p}{\left(f_{e}\right)}_{t}\right)}\right]$$

Where AUCR refers to the area under the concentration-time curve ratio that is the ratio of the levels of the object drug in the presence and absence of a drug interaction precipitant, *n* refers to the number of metabolizing enzymes potentially involved in a given interaction, *p* represents the number of intestinal efflux transporters, *m* represents the reversible inhibiting precipitants and/or metabolites, and *x* represents the time-dependent inhibiting precipitants and/or metabolites. [*I*]_*g*_ refers to the estimated gut enterocyte concentration of the precipitants, and [*I*]_*h*_ is the unbound hepatic inlet counterpart of the precipitant or metabolite. *K*_i_ is a precipitant’s or metabolite’s reversible inhibition constant; *k*_deg_, *k*_inact_, and *K*_I_ are the metabolizing enzymes’ time-dependent inhibition parameters and respectively describe the inhibited enzyme’s intrinsic degradation rate constant (property of the enzyme), the maximal inactivation rate constant (property of the precipitant or metabolite), and the time-dependent inhibition constant (for the precipitant or metabolite). Precipitant induction parameters include *d*, the induction scaling factor; maximum effect/response (*E*_max_) as induction’s maximal effect; and EC_50_, the precipitant concentration effecting half-maximal induction. *F*_g_, *f*_m_, and *f*_e_ are the fractions of the object drug escaping intestinal metabolism, the fraction undergoing hepatic metabolism, and the fraction of the object drug undergoing efflux transport by intestinal transporters.

For intestinal metabolism, where applicable, we only considered the effect of CYP3A4 and excluded other enzymes. We used the models of [*I*]_*g*_ described by Rostami-Hodjegan and Tucker^19^ (eq. 2) and [*I*]_*h*_ by Ito et al^20^ (eq. 3) as our precipitant concentrations of choice. In the case of metabolites, however, we calculated their hepatic inlet concentrations as their unbound *C*_max_ ($f_{u,p}\times C_{\max}$).

(2) $$\begin{array}{c} {\left[I\right]}_{g}=\frac{F_{a}\times k_{a}\times Dose}{Q_{en}} \end{array}$$

Where *F*_a_ is the fraction absorbed of the precipitant, *k*_a_ is the precipitant’s absorption rate constant, and *Q*_en_ is the quantified blood flow through enteric tissue per unit time (18 L/h/70 kg^21^).

(3) $$\begin{array}{c} {\left[I\right]}_{h}=f_{u,p}\times \left(C_{\max}+\frac{\left(F_{a}\times F_{g}\times k_{a}\times Dose\right)}{Q_{h}\times R_{B}}\right) \end{array}$$

Where *f*_u,p_ is the fraction of the precipitant drug (a drug precipitating a drug interaction) unbound to plasma proteins, *F*_g_ is the fraction of the precipitant drug escaping intestinal metabolism, *R*_B_ is the ratio of its blood-to-plasma distribution, and *Q*_h_ is the quantified blood flow through hepatic tissue per unit time (97 L/h/70 kg^21^).

In dabigatran etexilate models where carboxylesterases (CESs) may be reversibly inhibited, the modeled interactions included the potential inhibition of only P-gp and CES enzymes. To allow the models to reflect changes in active dabigatran levels as opposed to parent dabigatran etexilate, we multiplied the transporter component of equation 1 by the remaining working capacity of CES enzymes following their inhibition. The effect of inhibitory metabolites was incorporated when applicable. To further simplify this model, we considered CES1 and CES2 as a single pathway.

(4) $$\begin{array}{c} {AUCR}_{active\_dabigatran}={\left[\frac{1}{{\left(\frac{1}{1+\frac{{\left[I\right]}_{g}}{K_{i}}}\times {\;f}_{e}\right)}_{t}+\left(1-f_{e}\right)}\right]}_{P-gp}\times {\left[\frac{1}{1+\sum_{b=1}^{m}{\left(\frac{{\left[I\right]}_{h}}{K_{i}}\right)}_{b}}\right]}_{CES} \end{array}$$

### 2.3 Step 1: Estimating the role of BCRP and P-gp

To estimate the fractions of DOACs undergoing BCRP- and P-gp efflux transport (*f*_e_) in the intestine, we performed a model-based sensitivity analysis (step 1), where we fitted our model to clinical observations from DOAC clinical trials with index inhibitors. The analysis used equation 1 to simulate 9 drug interaction clinical trials that tested clarithromycin, erythromycin, and ketoconazole with DOACs (Supplemental Table 1). The 3 precipitants in this step were selected based on their ability to inhibit DOAC-metabolizing enzymes and transporters to differing degrees (Supplemental Table 7). This allowed the estimation of apparent *f*_e_ values, which simulate the clinical disposition of DOACs.

In each of the 9 simulations, all parameters were substituted into the model except *f*_e_. Each simulation was then equated to the corresponding clinical observation, and the model’s *f*_e_ of the object drug was tuned to the highest accuracy and least bias in reproducing the observed clinical effect. We used the root mean of squared errors as a measure of model accuracy, the geometric mean of fold errors (GMFE) for bias,^15^^,^^22^^,^^23^ and the 100% remainder of absolute bioavailabilities (${100\%-F}_{abs}$) of DOACs as constraints to the unidentified *f*_e_ values (Supplemental Table 2). In all iterations of this exercise, the sum of fractions transported by BCRP and P-gp equaled the defined constraints ($f_{e\_BCRP}+f_{e\_P-gp}=100\%-F_{abs})$. Because of the absence from the available literature, *F*_a_, *F*_g_, and *F*_H_ were set to 100%, respectively, assuming negligible loss of the object drug caused by absorption, gut metabolism, and hepatic first-pass effect.

(5) $$\begin{array}{c} RMSE=\sqrt{\sum_{r=1}^{n}\frac{{\left(r\right)}^{2}}{n}} \end{array}$$

Where *r* is the error calculated as the difference between a predicted and an observed value,

(6) $$\begin{array}{c} GMFE=\sqrt[{n}]{\prod_{i=1}^{n}10^{\left|\log\;f\right|}} \end{array}$$

and *f* is the fold error calculated as the ratio of a predicted value to an observed value.

### 2.4 Step 2: f_e_ estimates validation

Following the estimation of apparent *f*_e_ values, we validated our estimates by measuring their ability to reproduce 28 clinical trial observations of 13 precipitants with DOACs (Supplemental Table 3) (step 2). To simulate the trials, we used the same model described above (eq. 1), and to validate our estimates against trial observations, we used the same measures of accuracy and bias as in step 1. Root mean squared errors = 0 denoted the highest accuracy, and GMFE <2 was our cutoff for acceptable bias. Besides the 28 observations, 22 trial observations were excluded for using DOACs in microdoses.

The validated *f*_e_ estimates were then used to predict worst-case DOAC interaction signals with a select panel of precipitants.

### 2.5 Step 3: Prediction of worst-case drug interaction signals

In this chapter (step 3), we modeled a select group of drugs as potential precipitants affecting the levels of apixaban, dabigatran, edoxaban, and rivaroxaban. The precipitants were selected based on their recently confirmed status as BCRP inhibitors in vitro,^24^ in addition to their previously known in vitro inhibition of P-gp or CYP3A4, or both.^25^ Where applicable, metabolites that inhibited any DOAC-metabolizing enzyme were included in the simulations.

The same model described in step 1 above—excluding the enzyme induction component—was used to screen DOACs for drug interaction signals with the 47 drugs. We excluded enzyme induction to give worst-case estimates of the predicted signals.

### 2.6 Model input variables

Aside from the apparent *f*_e_ variables which were derived and validated in steps 1 and 2, the following sections define other model input variables included in steps 1, 2, and 3.

### 2.7 Precipitants’ input variables

Precipitants’ *C*_max_, enzyme, and transporter kinetics (*K*_i_, *K*_I_, *k*_inact_, *E*_max_, EC_50_) were collected from the DIDB. For *C*_max_, in steps 1 and 2, priority was given to clinical trials in healthy volunteers where the precipitants were administered in the same doses as the clinical trials (Supplemental Table 3). In step 3, however, the precipitant *C*_max_ was selected to correspond to the highest Food and Drug Administration–approved maintenance dose.

In steps 1, 2, and 3, the effect of metabolites was deliberately limited to hepatic metabolizing enzymes, assuming their formation following the parent drug’s metabolism and therefore their limited access to intestinal metabolizing enzymes and transporters. Additionally, in all steps, when metabolite concentrations were measured following a dose other than the precipitant dosing described above, their concentrations were extrapolated to correspond with the selected precipitant dose, assuming linear pharmacokinetics. Finally, when metabolite concentrations were not measured in humans, animal data describing the metabolite-to-parent ratio were used in the extrapolations. Otherwise, the effect of metabolites was not included when lacking both *C*_max_ and data supporting their extrapolation.

When selecting enzyme and transporter kinetics variables, priority was given to in vitro experiments using regulator-recommended substrates and enzyme systems when applicable.^21^^,^^26^^,^^27^ When multiple reports were made of the same in vitro kinetics, we calculated the median as a measure of centrality. We additionally halved the IC_50_ as a substitute for *K*_i_ when *K*_i_ was not reported. In dabigatran etexilate models where CES1 and CES2 were involved, to simplify the model, we modeled only the CES enzyme with the least inhibition. This combined the 2 enzymes in a single pathway, showing the least inhibition and the highest conversion of dabigatran etexilate into active dabigatran.

In enzyme induction models, we used protein increase or mRNA increase in place of *E*_max_ when a measured *E*_max_ was not reported.

Other physiological variables (ie, *Q*_en_, *Q*_h_), the enzyme induction scaling factor (*d*), and the fractions of drugs escaping gut metabolism (*F*_g_) were set to their regulator defaults.^21^^,^^27^ Absorption constants (*F*_a_, *K*_a_), enzyme degradation constants (*k*_deg_), and distribution constants (*R*_B_, *f*_u,p_) were collected from published literature. When unavailable in the literature, *f*_u,p_ of the metabolite was assumed to equal that of the parent drug. Specifically, however, in step 3, we used the highest recommended^21^ absorption parameters (*K*_a_ = 6 1/h, *F*_a_ =1) for the precipitants, and we assumed *F*_g_ as 1 as a measure for predicting worst-case drug interaction signals. Detailed references of the precipitant variables used in this study can be seen in the supplementary material.

### 2.8 Objects’ input variables: f_m_, and F_g_

In addition to the 2 transporters (BCRP and P-gp), the 4 object drugs were collectively identified as substrates of 5 metabolizing enzymes.^3^, ^4^, ^5^, ^6^ These were CYP3A4, CYP2J2, CYP1A2, CES1, and CES2. The fractions metabolized (*f*_m_) by each enzyme were gathered from the Food and Drug Administration sponsor reviews.^3^, ^4^, ^5^, ^6^ In contrast, the objects’ *F*_g_ variables were not reported in any literature available to the authors and were assumed as *F*_g_ = 1 throughout this study.^21^

## 3 Results

### 3.1 Estimation and validation of the fractions transported of DOACs

Dabigatran etexilate was estimated to have the greatest apparent *f*_e_ by intestinal P-gp (*f*_e_ = 0.61) (Table 1).^3^, ^4^, ^5^, ^6^ The same transporter was estimated to transport edoxaban to a lesser extent (*f*_e_ = 0.38), whereas it was shown to have the least effect on both apixaban and rivaroxaban (*f*_e_ = 0.21 for each drug). BCRP was also predicted to play an equal yet lesser role than P-gp in the efflux of apixaban and rivaroxaban (*f*_e_ = 0.13), whereas it played a minimal role in the efflux of edoxaban (*f*_e_ < 0.01). Validating the estimated fractions in step 2, edoxaban’s validations reproduced clinical observations with the highest accuracy and least bias (Table 2). This was followed by rivaroxaban and apixaban, whereas dabigatran etexilate’s *f*_e_ estimates showed the least accuracy and highest bias of all validations. All validations, except 1 pair, nonetheless, fell within acceptable GMFE limits (GMFE < 2) with 21 of the 28 validations showing a 0.8–1.25-fold error. Rifampin with dabigatran etexilate, however, predicted an interaction ratio 4 times the clinically observed interaction (Fig. 1).

**Table 1:** The estimated apparent fractions of DOACs undergoing efflux transport by intestinal P-gp and BCRP (step 1), with appended fractions metabolized collected from the Food and Drug Administration’s drug pharmacology reviews^3^, ^4^, ^5^, ^6^

Object Drug	Apparent *f*_e_		*f*_m_				
	P-gp	BCRP	CYP3A4	CYP2J2	CYP1A2	CES1	CES2
	*%*						
Apixaban	21	13	20.00	7.6^b^	7.6^b^	–	–
Dabigatran etexilate	61	–	–	–	–	7.0^b^	7.0^b^
Edoxaban	38	0	4.0^a^	–	–	10.0	–
Rivaroxaban	21	13	18.0	14.0	–	–	–

a Percentage includes the effect of CYP3A5 as opposed to only CYP3A4.

b Fractions reported in the literature as the total effect of 2 enzymes were assumed to equal the individual fraction metabolized by each of the 2 enzymes as worst-case estimates.

**Table 2:** Model validation outcomes using RMSE and GMFE as measures of accuracy and bias, respectively. Results arranged in order of decreasing accuracy and increasing bias (step 2)

Object Model	Measures of Accuracy and Bias	
	RMSE	GMFE
Edoxaban	0.136	1.069
Rivaroxaban	0.313	1.168
Apixaban	0.325	1.256
Dabigatran etexilate/dabigatran	0.558	1.368

RMSE = root of mean squared error.

**Fig. 1:**
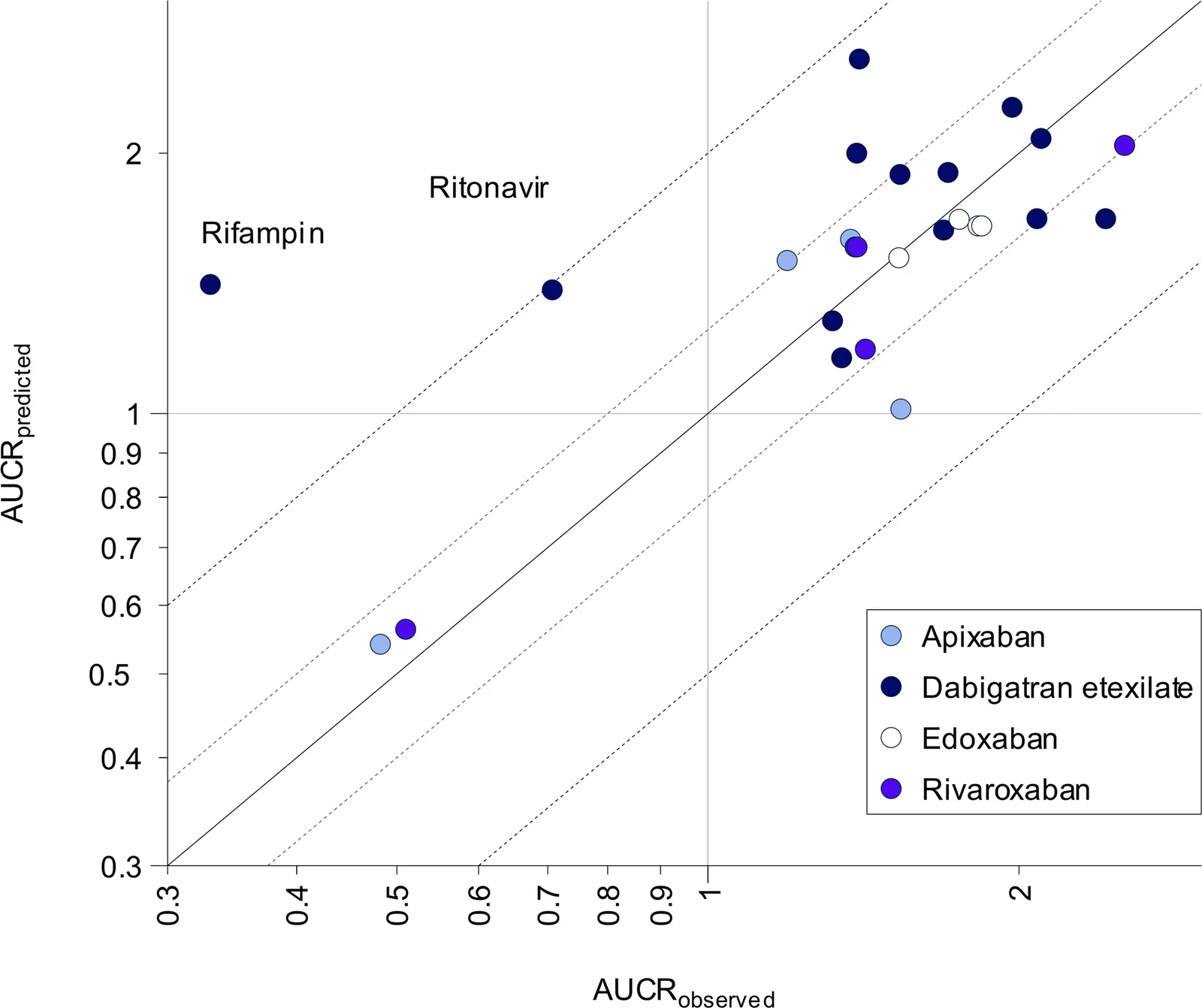
Model validations in step 2 (*n* = 28) comparing predictions using our estimated fractions of DOACs undergoing efflux by intestinal transporters to observed clinical values plotted as a logarithmic scale scatter plot where the solid line defines a fold error = 1; the inner dotted lines are the fold error limits of 0.8–1.25; and the outer dotted lines are the fold error limits of 0.5–2.

### 3.2 Drug interaction signal prediction

Forty-seven precipitants were included in the signal screening with DOACs, where 6 had metabolites potentially affecting DOAC hepatic metabolism. This returned 188 individual predictions of which one simulation predicted a DOAC plasma area under the concentration-time curve decrease <0.8, and 143 simulations predicted a DOAC plasma area under the concentration-time curve increase >1.25-fold, where 31 were >2-fold (Fig. 2).

**Fig. 2:**
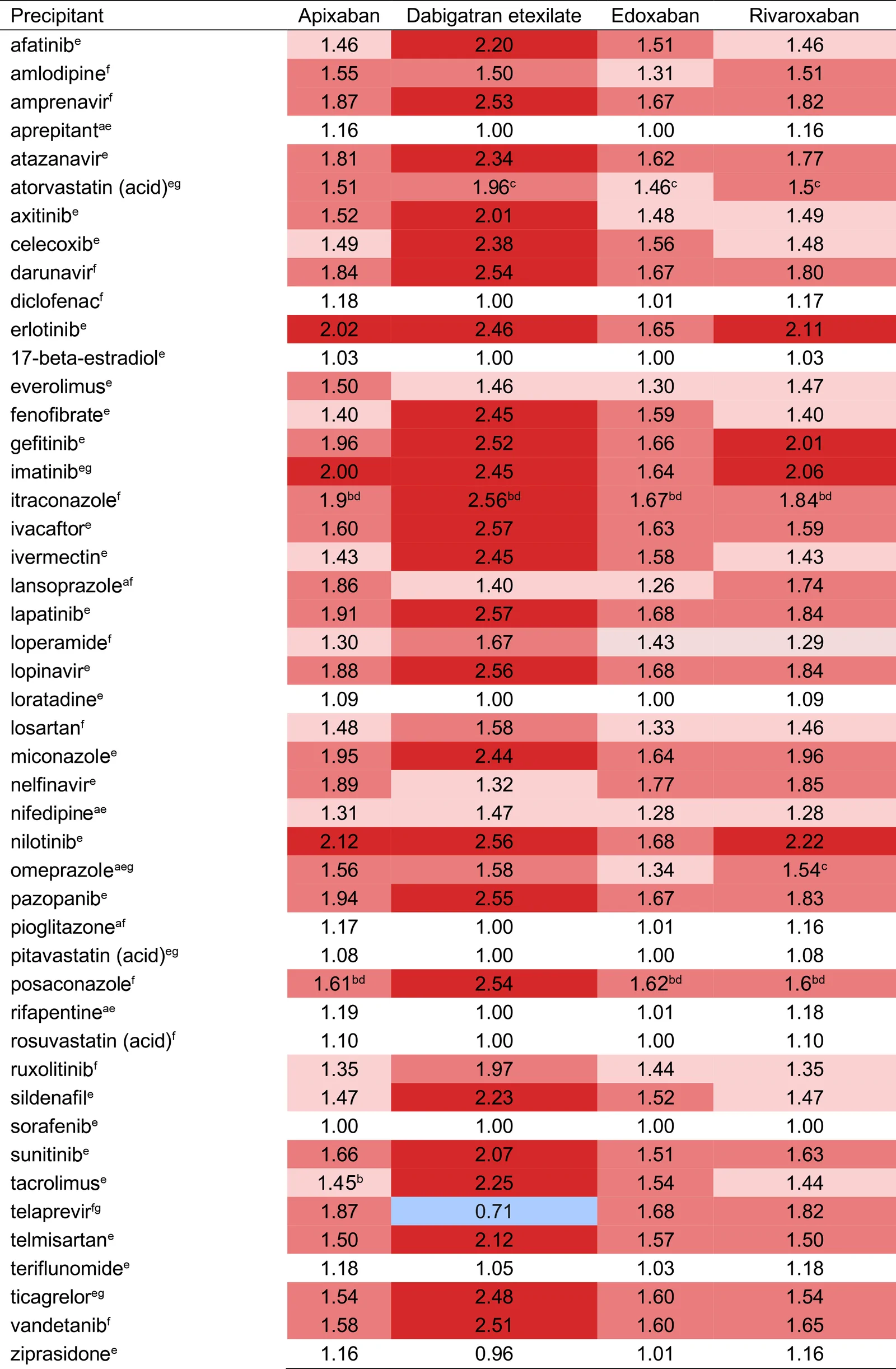
Drug interaction signals as predicted worst-case changes in the area under the concentration-time curve (AUC) of DOACs using inhibition only^*a*^ models and the highest precipitants' absorption parameters (*K*_a_, *F*_a_, and *F*_g_) for worst-case predictions (step 3). ^*a*^Precipitants known as CYP3A4 and/or CYP1A2 inducers; ^*b*^observed inhibition in clinical trials; ^*c*^no observed inhibition in clinical trials; ^*d*^trial used the object drug in microdose; ^*e*^precipitants designated as strong BCRP inhibitors in vitro by Deng and colleagues^24^; ^*f*^precipitants designated as moderate BCRP inhibitors in vitro by the same authors; ^*g*^precipitant has metabolites with action on relevant DOAC hepatic metabolizing enzymes. Signals are stratified according to their magnitude into <0.8 (blue), <1.25 (white), >1.25 (light red), >1.5 (medium red), and >2 (dark red),

Eleven precipitants showed no potential pharmacokinetic interaction signals with any of the tested DOACs (AUCR < 1.25). These were aprepitant, diclofenac, 17-*β*estradiol, loratadine, pioglitazone, pitavastatin acid, rifapentine, rosuvastatin acid, sorafenib, teriflunomide, and ziprasidone (Fig. 2). Supplemental Table 8 shows the estimated AUCRs as functions of inhibiting individual enzymes and transporters.

Analyzing our predictions’ precipitants by therapeutic group, the most precipitants belonging to the same group were 12 anticancer kinase inhibitors. They were followed by a diverse group of cardiovascular agents (*n* = 9), a group of anti-infective agents (*n* = 6), and 5 protease inhibitors. Of these groups, the tested kinase inhibitors had 44 potentially significant signals, of which 16 were >2-fold (Fig. 3).

**Fig. 3:**
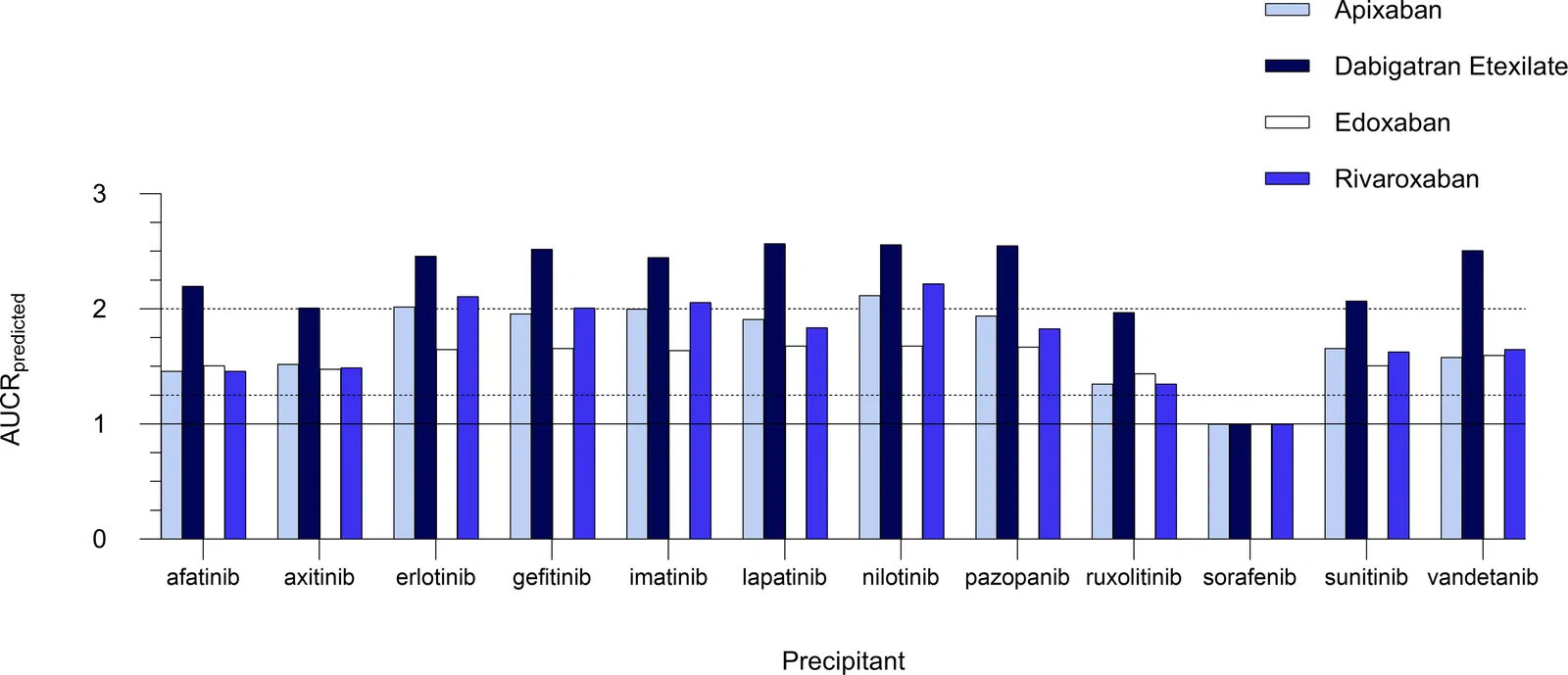
Drug interaction signal predictions (step 3) of 12 kinase inhibitors as potential inhibitors of DOAC transport and metabolism. Dotted lines indicate >1.25- and >2-fold increase in DOAC area under the concentration-time curve.

In the predictions where metabolites were considered, metabolites contributed minimally to the overall signals (<0.2% contribution to any predicted AUCR), and parent compounds were mainly responsible for any potential interaction. Additionally, the inhibition of intestinal CYP3A4 was not included in any simulation, because of the limited literature reporting *F*_g_ for our study’s precipitants.

## 4 Discussion

In our 3-step approach, we estimated the apparent *f*_e_ of 4 DOACs by 2 main intestinal efflux transporters. We derived our estimates using 9 clinical observations, and we validated them against 28 clinical trials. We then predicted the worst-case estimates of 188 drug-DOAC interactions where complex mechanisms involving both transporters and metabolizing enzymes were considered. Of our 188 signals, 144 predicted potentially significant drug interactions (area under the concentration-time curve change > 25%).

In our combined model, we included the reversible inhibition of intestinal BCRP and intestinal P-gp, intestinal CYP3A4, and relevant hepatic drug-metabolizing enzymes, and the time-dependent inhibition of the same enzymes. Even though our model (eq. 1) could accommodate multiple gut-metabolizing enzymes, in practice, however, the inclusion of intestinal CYP3A4 was limited by the lack of an experimentally measured *F*_g_. Because of the lack of a validated transporter induction model for BCRP and P-gp, only enzyme induction was included. Furthermore, enzyme induction was only considered in steps 1 and 2 to better match the clinical effects of precipitants when estimating and validating apparent *f*_e_ values. In practice, this was only relevant for rifampin in step 2. In step 3, however, enzyme induction was excluded because of the absence of several induction parameters from the DIDB. This coincided with our aim to predict worst-case estimates of potential drug interaction signals and to simplify high-throughput drug-drug interaction (DDI) screening, which we accomplished by excluding induction components.

In all 3 steps of this study, we assumed changes in the levels of dabigatran etexilate to correspond with those of active dabigatran. This was because, under physiological conditions, only 7% of absorbed dabigatran etexilate is converted by CES1 and CES2 to dabigatran, comprising 100% of the active drug. Any changes in the levels of dabigatran etexilate, assuming adequate CES activity, should therefore correspond with changes in active dabigatran levels, given the parent drug’s minimal bioavailability. This assumption was supported by clinical pharmacogenetic evidence showing decreased prodrug activation in carriers of CES decreased function alleles.^28^^,^^29^

In our validations (step 2), dabigatran etexilate’s apparent *f*_e_P-gp_ showed the least validation scores in reproducing the effects of rifampin on dabigatran etexilate (Fig. 1 and Supplemental Table 4). This was likely due to the lack of a transporter induction model and the model’s inability to predict the known P-gp induction by the same drug.^30^ This demonstrates that our model can be directionally wrong for known transporter inducers. In the same step, 22 clinical trial observations were excluded from *f*_e_ validations for using DOACs in microdoses or for lacking a suitable *C*_max_ of the precipitant (Supplemental Table 10). Where applicable, we included these trials in our prediction step, where most of our predicted signals (step 3) agreed with the included trial observations (*n* = 9). This includes an omeprazole–rivaroxaban trial, which showed no clinical inhibition but 1.54 AUCR in our predictions. We suspect that the model–excluded but previously known CYP3A4 and CYP1A2 induction played a role in amplifying our predictions^31^ (Supplemental Table 9). For these reasons, we consider our predictions of known transporter and/or enzyme inducers to be unreliable, without the inclusion of an induction model. Specifically, these were the predictions of aprepitant, lansoprazole, nifedipine, omeprazole, pioglitazone, rifampin, and rifapentine as inducers of CYP3A4; and omeprazole, rifabutin, and rifampin as inducers of CYP1A2; all of which lacked experimental induction variables in the DIDB.

Among the different precipitant therapeutic groups in our signal predictions (step 3), anticancer kinase inhibitors were the largest group. A scarcity of clinical data is available for our tested kinase inhibitors, having pharmacokinetic DDIs with DOACs. In the only available clinical trial, the authors concluded that the study was limited by a small sample size.^32^ Potential kinase inhibitor-DOAC interactions may be of special relevance, however, given the growing evidence and guidelines promoting the use of DOACs in the prophylaxis and treatment of cancer-associated thromboembolism.^33^, ^34^, ^35^, ^36^, ^37^ This relevance is the product of both the in vitro–confirmed CYP3A and P-gp inhibition by several kinase inhibitors^38^^,^^39^; and current cancer-associated thromboembolism guidelines, which generally recommend against the use of DOACs with strong CYP3A and/or P-gp inhibitors.^33^, ^34^, ^35^ Identifying such clinical interactions is therefore key to avoiding anticancer–DOAC interactions, which may increase bleeding risks in a vulnerable group of patients.

In contrast to the lack of clinical interaction studies with DOACs, some of our kinase inhibitors (step 3) have been the subjects of recent modeling work.^40^^,^^41^ Lafaie et al^41^ tested 11 drugs in vitro for P-gp–mediated interactions with apixaban and rivaroxaban. Six of the 11 drugs were in common with our studied kinase inhibitors. The authors used the basic model *I*_gut_/IC_50_ >10 to predict potentially significant DDI signals. Consistent with our findings, erlotinib, imatinib, lapatinib, and nilotinib showed signals for potential interactions with both apixaban and rivaroxaban. In contrast, however, our simulations for axitinib and sunitinib predicted an average increase of 57% in the AUCR of apixaban and rivaroxaban, whereas Lafaie et al^41^ suggested low probabilities of interaction for the same combinations (*I*_gut_/IC_50_ < 10).

In another work, Jin et al^40^ statically modeled axitinib, erlotinib, and gefitinib with apixaban, edoxaban, and rivaroxaban for CYP3A-mediated interactions. Compared with our study, CYP3A was included as a greater *f*_m_ of apixaban and rivaroxaban, and a lesser fraction in the case of edoxaban. Despite these differences, and the authors not considering transporters, Jin et al’^40^ predicted signals were nonetheless consistent with our findings.

Our predictive model is a combination of several previous models.^15^, ^16^, ^17^, ^18^^,^^22^ A main strength is its consideration of multiple interaction mechanisms and multiple proteins that may be involved in the disposition of an object drug. Additionally, it includes the effects of precipitant metabolites on hepatic metabolism. Given our validations’ agreement with clinical observations (step 2) and our predictions (step 3) with 9 clinical observations (Supplemental Table 9), as well as recent modeling work, this research builds confidence in static mechanistic models as a DDI prediction tool. Our approach may have also helped characterize the role of intestinal efflux transporters in DOAC pharmacokinetics. All of this consolidates the position of static mechanistic models in the prediction of DDI signals of other drug groups. It should be noted, however, that several limitations are associated with the same models and should therefore be considered when interpreting our results.

One such limitation is that our model could only include mechanistic components known, from in vitro evidence, to affect the pharmacokinetics of DOACs. Unrecognized or unquantified mechanisms were therefore not considered. This especially applies to our apparent *f*_e_ estimates, where we assumed intestinal P-gp and BCRP to be the only players in DOAC absorption (step 1). Our estimates may have consequently overestimated the transporters’ clinical role at the expense of other unknown mechanisms, which potentially affect DOACs’ absorption, namely *F*_a_, *F*_g_, and *F*_H_. This is especially applicable in the cases of apixaban, edoxaban, and rivaroxaban, where the sum of our assumed *f*_e_ estimates for both transporters always equals the 100% remainder of absolute bioavailability (${100\%-F}_{abs}$). In these cases, we predict a true clinical efflux fraction by BCRP and P-gp to be lower than our apparent estimates. The same limitation, however, did not apply to dabigatran etexilate, where only P-gp was considered. This is shown in our *f*_e_ which was estimated lower than ${100\%-F}_{abs}$. Despite this limitation, in the absence of experimentally measured *F*_a_, *F*_g_, and *F*_H_ variables, our *f*_e_ estimates conform to the physiological bioavailability relationship: ${100\%}_{dose}-f_{e_{total}}\geq F_{abs}\text{.}$

An added limitation is static models’ capacity to incorporate a single, static precipitant concentration as opposed to a dynamic range. This limits their representation of any drug’s concentration-time relationship, and together with their limited number of physiological variables, may limit their applicability to different disease states and patient populations. Nevertheless, this trade of complexity for a simplified model is likely what distinguishes static mechanistic models as a tool for detecting large numbers of drug interaction signals.

In conclusion, this work has provided methods for estimating the approximate contribution of intestinal efflux transporters in DOAC DDIs by combining comprehensive static mechanistic modeling equations with established clinical observations. We also predicted worst-case drug interaction signals involving DOACs and a diverse group of precipitants that included select kinase inhibitors. A large number of our signals warrant further characterization and quantification to evaluate their clinical actionability.

## Conflict of interest

The authors declare no conflicts of interest.
