# Supplementary material for: A 3-step static mechanistic modeling approach to predicting transporter- and enzyme-mediated interaction signals of direct oral anticoagulants
Source: Drug Metab Dispos. 2026 Jul 6;54(8):100360. doi: 10.1016/j.dmd.2026.100360 (PMC13688972; doi:10.1016/j.dmd.2026.100360)
Supplement: Supplementary file 1 [file mmc1.docx]

SUPPLEMENTARY INFORMATION

A Three-Step Static Mechanistic Modelling Approach to Predicting Transporter- and Enzyme-Mediated Interaction Signals of Direct Oral Anticoagulants

Mostafa ben Ahmed^1,2^, Anne M. Filppula^3^, Aleksi Tornio^1,2^

^1^Integrative Physiology and Pharmacology, Institute of Biomedicine, University of Turku, Turku, Finland.

^2^Unit of Clinical Pharmacology, Turku University Hospital, Turku, Finland.

^3^Pharmaceutical Sciences Laboratory, Department of Natural and Health Sciences, Faculty of Science and Engineering, Åbo Akademi University, Turku, Finland.

Contents

[Supplemental Table 1. Summary of the clinical observations used in the estimation of the fractions of DOACs undergoing efflux by BCRP and P-gp.	3](#_Toc229999565)

[Supplemental Table 2. Summary of object drug transporter variables’ constraints.	4](#_Toc229999566)

[Supplemental Table 3. Summary of the clinical observations used in the model validation (*step 2*).	5](#_Toc229999567)

[Supplemental Table 4. Validation simulations compared to observed clinical interactions.	7](#_Toc229999568)

[Supplemental Table 5. Precipitant and metabolite concentrations and their fractions unbound to plasma proteins used in *step 3*.	9](#_Toc229999569)

[Supplemental Table 6. Precipitants’ absorption and distribution kinetics relevant to the study’s static mechanistic models.	11](#_Toc229999570)

[Supplemental Table 7. Precipitants’ enzyme and transporter modulation kinetics collected from the DIDB.	14](#_Toc229999571)

[Supplemental Table 8. Single enzyme and transporter predictions as the effect of kinase inhibitors on the area under the concentration time curve of DOACs. For each DOAC, maximum attainable inhibition for each mechanism in the model is given for comparison.	24](#_Toc229999572)

[Supplemental Table 9*.* Summary of the drug Interaction clinical trial observations not included in model validation (*step 2*) and used to evaluate model predictions (*step 3*).	26](#_Toc229999573)

[Supplemental Table 10. Summary of the excluded microdose trials (step 2).	27](#_Toc229999574)

# Supplemental Table 1. Summary of the clinical observations used in the estimation of the fractions of DOACs undergoing efflux by BCRP and P-gp.

| Precipitant | Dose*^a^* (mg) | Interval*^a^* | C_max_*^a^ (*μM) | f_u,p_ | Object dose*^a^* (mg) | Observed DDI*^a^* (AUCR) |
| --- | --- | --- | --- | --- | --- | --- |
| Index precipitants used in the retrograde development of apixaban model variables | | | | | | |
| clarithromycin | 500 (7 days) | b.i.d. | 3.03 | 0.231*^b^* | 10 | 1.595 |
| 14-hydroxyclarithromycin |  |  | 2.37 | - |  |  |
| ketoconazole | 400 (6 days) | q.d. | 20.46 | 0.01*^c^* | 10 | 1.988 |
| N-deacetylketoconazole |  |  | 0.05 | 1*^d^* |  |  |
| Index precipitants used in the retrograde development of dabigatran etexilate model variables | | | | | | |
| ketoconazole | 400 (8 days) | q.d. | 16.68 | 0.01 | 150 | 2.530 |
| N-deacetylketoconazole |  |  | 0.04 | 1 |  |  |
| Index precipitants used in the retrograde development of edoxaban model variables | | | | | | |
| erythromycin | 500 (8 days) | q.i.d. | 1.67 | 0.1*^e^* | 60 | 1.855 |
| N-desmethylerythromycin |  |  | - | - |  |  |
| ketoconazole | 400 (7 days) | q.d. | 13.70 | 0.01 | 60 | 1.868 |
| N-deacetylketoconazole |  |  | 0.03 | 1 |  |  |
| Index precipitants used in the retrograde development of rivaroxaban model variables | | | | | | |
| clarithromycin | 500 (5 days) | b.i.d. | 3.41 | 0.231 | 10 | 1.540 |
| 14-hydroxyclarithromycin |  |  | 1.16 | - |  |  |
| erythromycin | 500 (6 days) | t.i.d. | 1.63 | 0.1 | 10 | 1.394 |
| N-desmethylerythromycin |  |  | - | - |  |  |
| ketoconazole | 200 (4 days) | q.d. | 6.37 | 0.01 | 10 | 1.820 |
| N-deacetylketoconazole |  |  | 0.01 | 1 |  |  |
| ketoconazole | 400 (5 days) | q.d. | 18.57 | 0.01 | 10 | 2.580 |
| N-deacetylketoconazole |  |  | 0.04 | 1 |  |  |

^a^ Data collected from the DIDB, and referencing clinical observations as reported by individual clinical trials; *^b^* (Traunmüller et al., 2007); *^c^* (Xu et al., 2018); *^d^* (Marok et al., 2023); *^e^* (FDA, n.d.). -, not available.

# Supplemental Table 2. Summary of object drug transporter variables’ constraints.

| Object drug | F_abs_ | Assumed f_e_ constraint | |
| --- | --- | --- | --- |
|  |  | P-gp | BCRP |
|  | *%* | *%* | *%* |
| apixaban | 66 *^a^* | 34 - f_e_BCRP_ | 34 - f_e_P-gp_ |
| dabigatran etexilate | 7 *^b^* | 93.0 | - |
| edoxaban | 62 *^c^* | 38 - f_e_BCRP_ | 38 - f_e_P-gp_ |
| rivaroxaban | 66 *^d^* | 34 - f_e_BCRP_ | 34 - f_e_P-gp_ |

F_abs_, absolute bioavailability; F_e_ constraints were assumed as the 100% remainder of individual DOACs’ absolute bioavailabilities. The above constraints assumed first-pass intestinal and hepatic metabolism to be negligible according to the relationship $F_{abs}=F_{a}\times F_{g}\times F_{h}$ where F_g_ and F_h_ are the fractions of DOACs escaping first-pass extraction in the intestine and liver respectively. *^a^*FDA. Drug Approval Package: ELIQUIS (apixaban) Application No.: 202155. December 28, 2012. Accessed April 17, 2024. <https://www.accessdata.fda.gov/drugsatfda_docs/nda/2012/202155orig1s000toc.cfm>; *^b^*FDA. Drug Approval Package: PRADAXA (dabigatran etexilate mesylate) Application No.: 022512. October 19, 2010. Accessed April 15, 2024. <https://www.accessdata.fda.gov/drugsatfda_docs/nda/2010/022512orig1s000toc.cfm>; *^c^*FDA. Drug Approval Package: SAVAYSA (edoxaban tosylate)Application No.: 206316. January 8, 2015. Accessed April 17, 2024. <https://www.accessdata.fda.gov/drugsatfda_docs/nda/2015/206316orig1orig2s000toc.cfm>; *^d^*FDA. Drug Approval Package: Xarelto (rivaroxaban) Application No.: 022406. July 1, 2011. Accessed April 17, 2024. <https://www.accessdata.fda.gov/drugsatfda_docs/nda/2011/022406Orig1s000TOC.cfm>

# Supplemental Table 3. Summary of the clinical observations used in the model validation (*step 2*).

| Precipitant | Precipitant Dose*^a^ (mg)* | Precipitant Interval*^a^* | C_max_*^a^* (μM) | f_u,p_ | Object Dose*^a^* (mg) | Observed DDI*^a^* (AUCR) |
| --- | --- | --- | --- | --- | --- | --- |
| Precipitants used in the validation of apixaban variables | | | | | | |
| cyclosporine | 100 (3 days) | q.d. | 0.55 | 0.037*^g^* | 10 | 1.193 |
| diltiazem | 360 (10 days) | q.d. | 0.73 | 0.22*^h^* | 10 | 1.374 |
| N-desmethyldiltiazem |  |  | 0.13 | 0.323*^d^* |  |  |
| N,N-didesmethyldiltiazem |  |  | - | - |  |  |
| naproxen (S-naproxen) | 500 | single dose | 319.74 | 0.003*^h^* | 10 | 1.537 |
| rifampicin | 600 (11 days) | q.d. | 11.81 | 0.175 | 10 | 0.482 |
| Precipitants used in the validation of dabigatran etexilate variables | | | | | | |
| clarithromycin | 500 (5 days) | b.i.d. | 3.41 | 0.231 | 300 | 1.969 |
| 14-hydroxyclarithromycin |  |  | 1.16 | - |  |  |
| clopidogrel | 600 | single dose | 0.01 | 0.02*^h^* | 150 mg (4 days) | 1.320 |
| clopidogrel | 300 | single dose | 0.01 | 0.02 | 150 mg (4 days) | 1.347 |
| clopidogrel acyl glucuronide |  |  | 2.77 | 0.01*^i^* |  |  |
| cobicistat | 150 (2 weeks) | q.d. | 2.39 | 0.025*^j^* | 150 | 2.100 |
| ibrexafungerp | 750 (4 days) | b.i.d. | 1.19 | 0.3*^k^* | 150 mg | 1.401 |
| rifampicin | 600 (8 days) | q.d. | 15.49 | 0.175 | 150 | 0.330 |
| ritonavir | 100 (3 doses) | b.i.d. | 2.26 | 0.02 | 75 mg | 1.691 |
| ritonavir | 100 (2 days) | b.i.d. | 2.26 | 0.02 | 75 mg | 1.690 |
| ritonavir | 100 (22 days) | q.d. | 2.28 | 0.02 | 150 | 0.707 |
| verapamil | 120 | single dose | 0.24 | 0.091 | 150 mg | 2.425 |
| verapamil | 120 | single dose | 0.24 | 0.091 | 150 mg | 2.081 |
| verapamil | 120 (4 days) | b.i.d. | 0.57 | 0.091 | 150 mg | 1.534 |
| verapamil | 120 (4 days) | q.i.d. | 0.97 | 0.091 | 150 mg | 1.393 |
| verapamil | 240 ER | single dose | 0.20 | 0.091 | 150 mg | 1.707 |
| Precipitants used in the validation of edoxaban variables | | | | | | |
| dronedarone | 400 (7 days) | b.i.d. | 0.30 | 0.02 | 60 mg (3 days) | 1.825 |
| dronedarone | 400 (7 days) | b.i.d. | 0.20 | 0.02*^h^* | 60 mg | 1.840 |
| N-desbutyldronedarone (SR35021) |  |  | 0.17 | 0.013*^l^* |  |  |
| quinidine | 300 | t.i.d. | 5.05 | 0.13*^h^* | 60 mg | 1.750 |
| verapamil | 240 SR | q.d. | 0.38 | 0.091 | 60 mg | 1.530 |
| Precipitants used in the validation of rivaroxaban variables | | | | | | |
| fluconazole | 400 (6 days) | q.d. | 64.23 | 0.89*^b^* | 20 mg single dose | 1.420 |
| rifampicin | 600 (7 days) | q.d. | 10.57 | 0.175*^c^* | 20 mg single dose | 0.510 |
| ritonavir | 600 (6 days) | b.i.d. | 19.93 | 0.02*^d^* | 10 mg single dose | 2.529 |
| ritonavir | 600 (6 days) | b.i.d. | 19.93 | 0.02 | 10 mg single dose | 2.530 |
| ritonavir M1 |  |  | - | - |  |  |
| verapamil | 360 (10 days) | q.d. | 0.57 | 0.091*^e^* | 20 mg single dose | 1.388 |
| verapamil | 360 (10 days) | q.d. | 0.97 | 0.091 | 20 mg free base single dose | 1.394 |
| norverapamil |  |  | 0.31 | 0.083*^f^* |  |  |
| N-dealkylverapamil |  |  | - | - |  |  |

*^a^* Data collected from the DIDB, and referencing clinical observations as reported by individual clinical trials.; NA not available in the DIDB; SR sustained release; Reference: *^b^* (L. Chen et al., 2022); *^c^* (Y. Xu et al., 2011); *^d^* (de Kanter et al., 2016); *^e^* (Neuhoff, Yeo, Barter, Jamei, Turner, & Rostami-Hodjegan, 2013); *^f^* (K.-F. Chen et al., 2020); *^g^* (Jamei et al., 2014); *^h^* (DIDB, 2023); *^i^* (Loer et al., 2022); *^j^* (FDA, 2012); *^k^* (Wring et al., 2017); *^l^* (FDA, 2009); -, not available.

# Supplemental Table 4. Validation simulations compared to observed clinical interactions.

| DOAC | Precipitant | AUCR_observed_ | AUCR_predicted_ | Fold error |
| --- | --- | --- | --- | --- |
| apixaban | cyclosporine | 1.19 | 1.5 | 1.26 |
| apixaban | diltiazem | 1.37 | 1.59 | 1.16 |
| apixaban | naproxen (S-naproxen) | 1.53 | 1.01 | 0.66 |
| apixaban | rifampin | 0.48 | 0.54 | 1.12 |
| dabigatran etexilate | clarithromycin | 1.96 | 2.26 | 1.14 |
| dabigatran etexilate | clopidogrel | 1.32 | 1.28 | 0.97 |
| dabigatran etexilate | clopidogrel | 1.34 | 1.16 | 0.86 |
| dabigatran etexilate | cobicistat | 2.1 | 2.08 | 0.98 |
| dabigatran etexilate | ibrexafungerp | 1.4 | 2.57 | 1.83 |
| dabigatran etexilate | rifampin | 0.33 | 1.41 | 4.27 |
| dabigatran etexilate | ritonavir | 1.69 | 1.63 | 0.96 |
| dabigatran etexilate | ritonavir | 1.69 | 1.63 | 0.96 |
| dabigatran etexilate | ritonavir | 0.7 | 1.39 | 1.96 |
| dabigatran etexilate | verapamil | 2.42 | 1.68 | 0.69 |
| dabigatran etexilate | verapamil | 2.08 | 1.68 | 0.8 |
| dabigatran etexilate | verapamil | 1.53 | 1.89 | 1.23 |
| dabigatran etexilate | verapamil | 1.39 | 2.00 | 1.43 |
| dabigatran etexilate | verapamil | 1.7 | 1.90 | 1.11 |
| edoxaban | dronedarone | 1.82 | 1.64 | 0.9 |
| edoxaban | dronedarone | 1.84 | 1.64 | 0.89 |
| edoxaban | quinidine | 1.75 | 1.67 | 0.95 |
| edoxaban | verapamil | 1.53 | 1.51 | 0.98 |
| rivaroxaban | fluconazole | 1.42 | 1.18 | 0.83 |
| rivaroxaban | rifampin | 0.51 | 0.56 | 1.09 |
| rivaroxaban | ritonavir | 2.52 | 2.04 | 0.8 |
| rivaroxaban | ritonavir | 2.53 | 2.04 | 0.8 |
| rivaroxaban | verapamil | 1.38 | 1.55 | 1.12 |
| rivaroxaban | verapamil | 1.39 | 1.55 | 1.11 |

# Supplemental Table 5. Precipitant and metabolite concentrations and their fractions unbound to plasma proteins used in *step 3*.

| Precipitant | Precipitant Dose (mg) | Precipitant Interval | C_max_ (μg/ml) | f_up_ | f_up_ reference |
| --- | --- | --- | --- | --- | --- |
| afatinib | 40 | q.d. | 0.046 | 0.095 | Bartelink et al. (2022) |
| amlodipine | 10 | q.d. | 0.0288 | 0.02 | Han et al. (2021) |
| amprenavir | 1200 | b.i.d. | 8.56 | 0.1 | DrugBank. Amprenavir |
| aprepitant | 80 | q.d. | 1.9 | 0.05 | Pepin et al. (2021) |
| atazanavir | 300 | b.i.d. | 4.817 | 0.14 | Montanha et al. (2022) |
| atorvastatin (acid) | 80 | q.d. | 0.0982 | 0.027 | Avvari et al. (2023) |
| axitinib | 5 | b.i.d. | 0.0417 | 0.01 | DrugBank. Axitinib. |
| celecoxib | 200 | b.i.d. | 1.4745 | 0.03 | Porat et al. (2023) |
| darunavir | 600 | b.i.d. | 7.97 | 0.05 | Dong et al. (2022) |
| diclofenac | 50 | t.i.d. | 1.284 | 0.003 | Dancik et al. (2023) |
| erlotinib | 150 | q.d. | 3.02 | 0.088 | Bartelink et al. (2022) |
| estradiol (17-beta-estradiol) | 1 | single dose | 0.053118 | 0.05 | DrugBank. Estradiol. |
| everolimus | 5 | q.d. | 0.0413 | 0.26 | DrugBank. Everolimus. |
| fenofibrate | 145 | q.d. | 12.9 | 0.01 | DrugBank. Fenofibrate |
| gefitinib | 250 | q.d. | 0.543 | 0.089 | Chen et al. (2017) |
| imatinib | 400 | b.i.d. | 4.245 | 0.05 | Centanni et al. (2024) |
| itraconazole | 200 | b.i.d. | 2.94 | 0.0015 | Udomnilobol et al. (2024) |
| ivacaftor | 150 | b.i.d. | 0.572 | 0.001 | Amiel et al (2024) |
| ivermectin | 12 | single dose | 0.057 | 0.0014 | Rowland Yeo and Wesche (2023) |
| lansoprazole | 30 | q.d. | 831.15 | 0.03 | Ezuruike et al. (2023) |
| lapatinib | 1250 | q.d. | 1.48 | 0.014 | Li et al. (2022) |
| loperamide | 16 | single dose | 0.00388755 | 0.05 | DrugBank. Loperamide. |
| lopinavir | 400 | b.i.d. | 7.29 | 0.02 | Perazzolo et al. (2021) |
| loratadine | 10 | q.d. | 0.0126 | 0.03 | Liu et al. (2024) |
| losartan | 100 | q.d. | 0.62799 | 0.014 | Tanveer et al. (2022) |
| miconazole | 85 | t.i.d. | 0.0833 | 0.01 | assumed based on: Miconazole - a ScienceDirect overview referencing Meyler's Side Effects of Drugs (Sixteenth Edition) |
| nelfinavir | 750 | t.i.d. | 3.03 | 0.014 | Peng et al. (2022) |
| nifedipine | 20 | b.i.d. | 0.151 | 0.04 | Romão et al. (2024) |
| nilotinib | 800 | single dose | 1.872 | 0.065 | Cheong et al. (2021) |
| omeprazole | 40 | q.d. | 1.85 | 0.05 | Boddu et al (2024) |
| pazopanib | 800 | q.d. | 73.3 | 0.001 | Centanni et al. (2024) |
| pioglitazone | 45 | q.d. | 1.96 | 0.005 | Adiwidjaja et al. (2024) |
| pitavastatin (acid) | 4 | q.d. | 0.077 | 0.0064 | Hedge and Morse (2024) |
| posaconazole | 300 | q.d. | 1.3 | 0.01 | Watanbe and Kotsuma (2024) |
| rifapentine | 900 | thrice weekly | 21.2 | 0.02 | Reali et al. (2023) |
| rosuvastatin (acid) | 40 | q.d. | 0.0352 | 0.107 | Adiwidjaja et al. (2024) |
| ruxolitinib | 20 | b.i.d. | 0.126 | 0.033 | Gerner et al. (2022) |
| sildenafil | 100 | single dose | 0.294 | 0.04 | Porat et al (2023) |
| sorafenib | 400 | b.i.d. | 11.9 | 0.009 | Wang et al. (2021) |
| sunitinib | 50 | q.d. | 0.0386 | 0.05 | Centanni et al. (2024) |
| tacrolimus | 10 | single dose | 0.0587 | 0.013 | Guan et al. (2024) |
| telaprevir | 1125 | b.i.d. | 2.323 | 0.41 | Kimoto et al. (2019) |
| telmisartan | 80 | q.d. | 1.5571 | 0.005 | Tsuchitani et al. (2024) |
| teriflunomide | 14 | q.d. | 45.2 | 0.00213 | Hopkins et al. (2015) |
| ticagrelor | 90 | b.i.d. | 1.033 | 0.002 | Wang et al. (2020) |
| vandetanib | 800 | single dose | 0.845 | 0.1 | Wang et al. (2022) |
| ziprasidone | 80 | single dose | 0.055 | 0.1 | Wagner et al. (2021) |
| *atorvastatin lactone* | 80 | q.d. | 0.0116 | 0.012 | Reig-Lopez et al., 2022 |
| N-desmethylimatinib (CGP74588) | 400 | b.i.d. | 0.588 | 0.036 | Loer et al., 2024 |
| 5-hydroxyomeprazole | 40 | q.d. | 0.26 | 0.17 | Lenoir et al., 2022 |
| *pitavastatin lactone* | 4 | q.d. | 0.0512 | - | - |
| telaprevir (R)-diastereomer VRT-127394 | 1125 | b.i.d. | 1.544 | - | - |
| deshydroxyethoxy ticagrelor | 90 | b.i.d. | 0.281 | - | - |

Italic text denotes metabolite concentrations extrapolated to correspond to the modelled precipitant concentration (see Model input variables in Methods). The extrapolation assumed linear pharmacokinetics. C_max_ reports were sourced from the DIDB. -, not available in the literature available to the authors.

# Supplemental Table 6. Precipitants’ absorption and distribution kinetics relevant to the study’s static mechanistic models.

| Precipitant | R_B_ | R_B_ reference | K_a_ | K_a_ reference | F_a_ | F_a_ reference |
| --- | --- | --- | --- | --- | --- | --- |
| afatinib | 1.27 | Bartelink et al. (2022) | 6 | Assumed | 1 | Assumed |
| amlodipine | 0.065 | Han et al. (2021) | 6 | Assumed | 1 | Assumed |
| amprenavir | 1 | Assumed | 6 | Assumed | 1 | Assumed |
| aprepitant | 0.86 | Pepin et al. (2021) | 6 | Assumed | 1 | Assumed |
| atazanavir | 0.75 | Montanha et al. (2022) | 6 | Assumed | 1 | Assumed |
| atorvastatin (acid) | 0.61 | Avvari et al. (2023) | 6 | Assumed | 1 | Assumed |
| axitinib | 1 | Assumed | 6 | Assumed | 1 | Assumed |
| celecoxib | 0.89 | Porat et al. (2023) | 6 | Assumed | 1 | Assumed |
| clarithromycin | 1.23 | Moj et al., 2017 | 1 | Xu et al., 2018 | 1 | Xu et al., 2018 |
| clopidogrel | 0.72 | Duong et al., (2022) | 1 | Duong et al., (2022) | 0.49 | Duong et al., (2022) |
| cobicistat | 0.589 | Grillo et al., 2012 | 6 | Assumed | 1 | Assumed |
| cyclosporine | 1.36 | de Kanter et al., (2016) | 1.14 | Jamei et al., (2014) | 1 | de Kanter et al., (2016) |
| darunavir | 0.64 | Dong et al. (2022) | 6 | Assumed | 1 | Assumed |
| diclofenac | 0.605 | Dancik et al. (2023) | 6 | Assumed | 1 | Assumed |
| diltiazem | 0.96 | de Kanter et al., 16 | 6 | de Kanter et al., 16 | 1 | de Kanter et al., (2016) |
| dronedarone | 1.87 | Ven Hui Leo et al., (2023) | 0.816 | Wen et al., (2022) | 0.898 | Wen et al., (2022) |
| erlotinib | 0.95 | Bartelink et al. (2022) | 6 | Assumed | 1 | Assumed |
| erythromycin | 0.854 | Grillo et al., 2012 | 0.26 | Grillo et al., 2012 | 1 | Grillo et al., 2012 |
| 17-beta-estradiol | 1 | Assumed | 6 | Assumed | 1 | Assumed |
| everolimus | 1 | Assumed | 6 | Assumed | 1 | Assumed |
| fenofibrate | 1 | Assumed | 6 | Assumed | 1 | Assumed |
| fluconazole | 1 | Chen et al., (2022) | 1.755176 | Jamei et al., (2014) | 0.9858426 | De Zwart et al., (2021) |
| gefitinib | 0.8 | Chen et al. (2017) | 6 | Assumed | 1 | Assumed |
| ibrexafungerp | 0.7 | Wring et al., (2017) | 6 | Assumed | 1 | Assumed |
| imatinib | 0.73 | Adattini et al. (2023) | 6 | Assumed | 1 | Assumed |
| itraconazole | 0.6 | Udomnilobol et al. (2024) | 6 | Assumed | 1 | Assumed |
| ivacaftor | 0.55 | t Amiel et al (2024) | 6 | Assumed | 1 | Assumed |
| ivermectin | 0.71 | Rowland Yeo and Wesche (2023) | 6 | Assumed | 1 | Assumed |
| ketoconazole | 1 | Perdaems et al. 2010 | 1.62 | Xu et al., 2018 | 1 | Xu et al., 2018 |
| lansoprazole | 0.59 | Ezuruike et al. (2023) | 6 | Assumed | 1 | Assumed |
| lapatinib | 0.7 | Li et al. (2022) | 6 | Assumed | 1 | Assumed |
| loperamide | 1 | Assumed | 6 | Assumed | 1 | Assumed |
| lopinavir | 0.58 | Perazzolo et al. (2021) | 6 | Assumed | 1 | Assumed |
| loratadine | 1 | Liu et al. (2024) | 6 | Assumed | 1 | Assumed |
| losartan | 1 | Assumed | 6 | Assumed | 1 | Assumed |
| miconazole | 1 | Assumed | 6 | Assumed | 1 | Assumed |
| S-naproxen | 1 | Assumed | 6 | Assumed | 1 | Assumed |
| nelfinavir | 1 | Peng et al. (2022) | 6 | Assumed | 1 | Assumed |
| nifedipine | 0.74 | Romão et al. (2024) | 6 | Assumed | 1 | Assumed |
| nilotinib | 0.71 | Cheong et al. (2021) | 6 | Assumed | 1 | Assumed |
| omeprazole | 0.75 | Boddu et al. (2024) | 6 | Assumed | 1 | Assumed |
| pazopanib | 0.55 | Wu et al (2022) | 6 | Assumed | 1 | Assumed |
| pioglitazone | 0.67 | Adiwidjaja et al. (2024) | 6 | Assumed | 1 | Assumed |
| pitavastatin (acid) | 0.55 | Hedge and Morse (2024) | 6 | Assumed | 1 | Assumed |
| posaconazole | 0.62 | Watanbe and Kotsuma (2024) | 6 | Assumed | 1 | Assumed |
| quinidine | 0.82 | Simcyp v23 | 3 | Simcyp v23 | 1 | Simcyp v23 |
| rifampin | 0.9 | Xu et al., (2011) | 0.51 | Xu et al., (2011) | 1 | de Kanter et al., (2016) |
| rifapentine | 1 | Reali et al. (2023) | 6 | Assumed | 1 | Assumed |
| ritonavir | 0.59 | de Kanter et al., (2016) | 0.24 | de Kanter et al., (2016) | 1 | de Kanter et al., (2016) |
| rosuvastatin (acid) | 0.63 | Adiwidjaja et al. (2024) | 6 | Assumed | 1 | Assumed |
| ruxolitinib | 1.2 | Alsmadi et al. (2021) | 6 | Assumed | 1 | Assumed |
| sildenafil | 0.81 | Porat et al (2023) | 6 | Assumed | 1 | Assumed |
| sorafenib | 0.59 | Wang et al. (2021) | 6 | Assumed | 1 | Assumed |
| sunitinib | 1.17 | Yu et al. (2020) | 6 | Assumed | 1 | Assumed |
| tacrolimus | 35 | Guan et al. (2024) | 6 | Assumed | 1 | Assumed |
| telaprevir | 0.65 | Kimoto et al. (2019) | 6 | Assumed | 1 | Assumed |
| telmisartan | 0.775 | Tsuchitani et al. (2024) | 6 | Assumed | 1 | Assumed |
| teriflunomide | 1 | Assumed | 6 | Assumed | 1 | Assumed |
| ticagrelor | 0.4 | Wang et al. (2020) | 6 | Assumed | 1 | Assumed |
| vandetanib | 1 | Wang et al. (2022) | 6 | Assumed | 1 | Assumed |
| verapamil | 0.705 | Neuhoff et al., (2013) | 1.22 | de Kanter et al., (2016) | 1 | de Kanter et al., (2016) |
| ziprasidone | 0.81 | Wagner et al. (2021) | 6 | Assumed | 1 | Assumed |

# Supplemental Table 7. Precipitants’ enzyme and transporter modulation kinetics collected from the DIDB.

| Precipitant | Enzyme/Transporter | Ki (μM) | KI (μM) | kinact (h^-1^) | Emax | EC50 |
| --- | --- | --- | --- | --- | --- | --- |
| afatinib | P-gp | 3.4 | - | - | - | - |
| afatinib | CYP3A4 | 53.38 | - | - | - | - |
| afatinib | BCRP | 0.38 | - | - | - | - |
| amlodipine | CYP3A4 | 17.84 | 3.3 | 2.34 | - | - |
| amlodipine | CYP2J2 | 2.58 | - | - | - | - |
| amlodipine | P-gp | 7 | - | - | - | - |
| amlodipine | CYP1A2 | 2.25 | - | - | - | - |
| amlodipine | BCRP | 7.84 | - | - | - | - |
| amprenavir | CYP3A4 | 1 | 0.64 | 27.00 | - | - |
| amprenavir | P-gp | 20 | - | - | - | - |
| amprenavir | BCRP | 91 | - | - | - | - |
| aprepitant | CYP3A4 | 10 | - | - | - | - |
| aprepitant | CYP1A2 | 50 | - | - | - | - |
| aprepitant | BCRP | 1.99 | - | - | - | - |
| atazanavir | P-gp | 19.08 | - | - | - | - |
| atazanavir | CYP3A4 | 0.425 | - | - | - | - |
| atazanavir | BCRP | 21.10 | - | - | - | - |
| atorvastatin (acid) | BCRP | 10.3 | - | - | - | - |
| atorvastatin (acid) | P-gp | 11.85 | - | - | - | - |
| atorvastatin (acid) | CYP3A4 | 92.75 | 29.9 | 1.14 | - | - |
| atorvastatin (acid) | CYP1A2 | 50 | - | - | - | - |
| axitinib | CYP3A4 | 8.3 | 0.93 | 0.822 | - | - |
| axitinib | CYP1A2 | 0.11 | - | - | - | - |
| axitinib | P-gp | 2 | - | - | - | - |
| axitinib | BCRP | 2.82 | - | - | - | - |
| celecoxib | CYP1A2 | 25.4 | - | - | - | - |
| celecoxib | P-gp | 19.23 | - | - | - | - |
| celecoxib | BCRP | 12.05 | - | - | - | - |
| clarithromycin | CYP3A4 | 21.35 | 13.86 | 2.25 | - | - |
| clarithromycin | P-gp | 7.55 | - | - | - | - |
| clarithromycin | CYP1A2 | 250.5 | - | - | - | - |
| clarithromycin | BCRP | 205.5 | - | - | - | - |
| clopidogrel | P-gp | 72.55 | - | - | - | - |
| clopidogrel | CYP3A4 | 3.0455 | 87.4 | 3.18 | - | - |
| clopidogrel | CYP1A2 | 12.15 | - | - | - | - |
| clopidogrel | CES1 | 12.35 | - | - | - | - |
| clopidogrel | BCRP | 31.5 | - | - | - | - |
| cobicistat | P-gp | 11.825 | - | - | - | - |
| cobicistat | CYP3A4 | 0.06 | - | - | - | - |
| cobicistat | BCRP | 29.5 | - | - | - | - |
| cyclosporine | P-gp | 0.46 | - | - | - | - |
| cyclosporine | CYP3A4 | 13.65 | 3.3 | 1.98 | - | - |
| cyclosporine | BCRP | 7.25 | - | - | - | - |
| darunavir | P-gp | 7.35 | - | - | - | - |
| darunavir | CYP3A4 | 0.4675 | - | - | - | - |
| darunavir | BCRP | 55 | - | - | - | - |
| diclofenac | CYP1A2 | 88 | - | - | - | - |
| diclofenac | CYP3A4 | 155.5 | 1640 | 15 | - | - |
| diclofenac | CYP2J2 | 25 | - | - | - | - |
| diclofenac | BCRP | 36 | - | - | - | - |
| diltiazem | CYP3A4 | 41 | 4.15 | 1.02 | - | - |
| diltiazem | CES2 | 7.215 | - | - | - | - |
| diltiazem | CES1 | 9 | - | - | - | - |
| diltiazem | P-gp | 22.65 | - | - | - | - |
| diltiazem | CYP2J2 | 25.5 | - | - | - | - |
| dronedarone | CYP3A4 | 5.82 | 0.87 | 2.34 | - | - |
| dronedarone | CYP2J2 | 0.482 | 0.0405 | 0.0275 | - | - |
| dronedarone | P-gp | 1.4875 | - | - | - | - |
| erlotinib | CYP3A4 | 1.28 | 8 | 3 | - | - |
| erlotinib | CYP2J2 | 0.76 | - | - | - | - |
| erlotinib | BCRP | 0.15 | - | - | - | - |
| erlotinib | P-gp | 3.98 | - | - | - | - |
| erlotinib | CYP1A2 | - | - | - | - | - |
| erythromycin | CYP3A4 | 66 | 13.18 | 2.37 | - | - |
| erythromycin | P-gp | 14.2 | - | - | - | - |
| erythromycin | CYP2J2 | 25.00 | - | - | - | - |
| erythromycin | CYP1A2 | 136.25 | - | - | - | - |
| erythromycin | BCRP | - | - | - | - | - |
| 17-beta-estradiol | CYP3A4 | 20 | - | - | - | - |
| 17-beta-estradiol | CYP1A2 | 26.5 | - | - | - | - |
| 17-beta-estradiol | BCRP | 5 | - | - | - | - |
| everolimus | CYP3A4 | 13 | 0.9 | 1.32 | - | - |
| everolimus | P-gp | 2 | - | - | - | - |
| everolimus | CES2 | 4.905 | - | - | - | - |
| everolimus | BCRP | 1.09 | - | - | - | - |
| fenofibrate | CES2 | 44.1 | - | - | - | - |
| fenofibrate | P-gp | 2.92 | - | - | - | - |
| fenofibrate | CYP3A4 | 71.05 | - | - | - | - |
| fenofibrate | CYP1A2 | 500 | - | - | - | - |
| fenofibrate | BCRP | 85 | - | - | - | - |
| fluconazole | CYP3A4 | 11.3 | - | - | - | - |
| fluconazole | CYP1A2 | 50.5 | - | - | - | - |
| gefitinib | CYP3A4 | 16 | 14.10 | 1.14 | - | - |
| gefitinib | CYP2J2 | 2.99 | - | - | - | - |
| gefitinib | P-gp | 2.70 | - | - | - | - |
| gefitinib | BCRP | 0.51 | - | - | - | - |
| gefitinib | CYP1A2 | - | - | - | - | - |
| ibrexafungerp | P-gp | 3.5 | - | - | - | - |
| ibrexafungerp | CYP3A4 | 5.55 | - | - | - | - |
| ibrexafungerp | BCRP | 5.75 | - | - | - | - |
| imatinib | CYP3A4 | 7.68 | 14 | 2 | - | - |
| imatinib | CYP2J2 | 3.53 | - | - | - | - |
| imatinib | P-gp | 19.05 | - | - | - | - |
| imatinib | CYP1A2 | 205 | - | - | - | - |
| imatinib | BCRP | 0.13 | - | - | - | - |
| itraconazole | CYP3A4 | 0.23 | 3.92 | 2.58 | - | - |
| itraconazole | P-gp | 0.90 | - | - | - | - |
| itraconazole | CYP1A2 | 5 | - | - | - | - |
| itraconazole | BCRP | 1.30 | - | - | - | - |
| ivacaftor | P-gp | 0.57 | - | - | - | - |
| ivacaftor | CYP3A4 | 2.15 | - | - | - | - |
| ivacaftor | BCRP | 3.57 | - | - | - | - |
| ivermectin | BCRP | 1.4 | - | - | - | - |
| ivermectin | P-gp | 0.15 | - | - | - | - |
| ivermectin | CYP3A4 | 5.375 | - | - | - | - |
| ivermectin | CYP1A2 | 5 | - | - | - | - |
| ketoconazole | P-gp | 0.96 | - | - | - | - |
| ketoconazole | CYP3A4 | 0.023 | - | - | - | - |
| ketoconazole | CYP2J2 | 0.082 | - | - | - | - |
| ketoconazole | CES2 | 181.2 | - | - | - | - |
| ketoconazole | CYP1A2 | 8.75 | - | - | 10 | - |
| ketoconazole | BCRP | 4.575 | - | - | - | - |
| ketoconazole | CES1 | 4.15 | - | - | - | - |
| lansoprazole | P-gp | 31.40 | - | - | - | - |
| lansoprazole | CYP3A4 | 1.75 | 31.8 | 4.38 | - | - |
| lansoprazole | CYP2J2 | 25 | - | - | - | - |
| lansoprazole | CYP1A2 | 4.05 | - | - | - | - |
| lansoprazole | BCRP | 7.20 | - | - | - | - |
| lapatinib | CYP3A4 | 2.47 | 4.6 | 1.74 | - | - |
| lapatinib | P-gp | 1.64 | - | - | - | - |
| lapatinib | CYP1A2 | 15 | - | - | - | - |
| lapatinib | BCRP | 0.03 | - | - | - | - |
| loperamide | P-gp | 4 | - | - | - | - |
| loperamide | BCRP | 16 | - | - | - | - |
| loperamide | CYP3A4 | 0.7 | - | - | - | - |
| loperamide | CYP1A2 | 18.75 | - | - | - | - |
| loperamide | CES2 | 1.215 | - | - | - | - |
| loperamide | CES1 | 0.332 | - | - | - | - |
| lopinavir | P-gp | 2.00 | - | - | - | - |
| lopinavir | CYP3A4 | 3.6 | 1 | 7 | - | - |
| lopinavir | BCRP | 6.05 | - | - | - | - |
| loratadine | CYP3A4 | 16 | - | - | - | - |
| loratadine | CYP1A2 | 32.5 | - | - | - | - |
| loratadine | BCRP | 5.45 | - | - | - | - |
| losartan | P-gp | 52 | - | - | - | - |
| losartan | CYP3A4 | 105 | 3.3 | 0.36 | - | - |
| losartan | CYP2J2 | 111.5 | - | - | - | - |
| losartan | CYP1A2 | 262 | - | - | - | - |
| losartan | BCRP | 9.95 | - | - | - | - |
| miconazole | CYP3A4 | 0.055 | - | - | - | - |
| miconazole | P-gp | 7.55 | - | - | - | - |
| miconazole | CYP2J2 | 0.29 | - | - | - | - |
| miconazole | CYP1A2 | 1.45 | - | - | - | - |
| miconazole | BCRP | 2.32 | - | - | - | - |
| S-naproxen | CES1 | 707 | - | - | - | - |
| S-naproxen | CYP2J2 | 25.5 | - | - | - | - |
| S-naproxen | P-gp | - | - | - | - | - |
| nelfinavir | CYP3A4 | 4.8 | 1.41 | 12.12 | - | - |
| nelfinavir | CYP1A2 | 190 | - | - | - | - |
| nelfinavir | CES1 | 3.7 | - | - | - | - |
| nelfinavir | P-gp | 0.70 | - | - | - | - |
| nelfinavir | BCRP | 6.50 | - | - | - | - |
| nifedipine | CYP3A4 | 20.00 | - | - | - | - |
| nifedipine | CYP2J2 | 5.08 | - | - | - | - |
| nifedipine | P-gp | 28.90 | - | - | - | - |
| nifedipine | CYP1A2 | 0.825 | - | - | - | - |
| nifedipine | CES1 | 10 | - | - | - | - |
| nifedipine | BCRP | 18 | - | - | - | - |
| nilotinib | CYP3A4 | 0.448 | 2 | 2 | - | - |
| nilotinib | CYP2J2 | 0.12 | - | - | - | - |
| nilotinib | BCRP | 0.69 | - | - | - | - |
| nilotinib | P-gp | 1.85 | - | - | - | - |
| nilotinib | CYP1A2 | 50 | - | - | - | - |
| omeprazole | CYP3A4 | 41.9 | 37 | 4 | - | - |
| omeprazole | CYP1A2 | 163 | - | - | - | - |
| omeprazole | P-gp | 26.00 | - | - | - | - |
| omeprazole | CYP2J2 | 25 | - | - | - | - |
| omeprazole | BCRP | 7.65 | - | - | - | - |
| pazopanib | P-gp | 4.03 | - | - | - | - |
| pazopanib | CYP3A4 | 6 | 3 | 1 | - | - |
| pazopanib | CYP1A2 | 8 | - | - | - | - |
| pazopanib | BCRP | 5.21 | - | - | - | - |
| pioglitazone | CYP3A4 | 11.8 | 13 | 1 | - | - |
| pioglitazone | BCRP | 28.6 | - | - | - | - |
| pitavastatin (acid) | BCRP | 2.92 | - | - | - | - |
| pitavastatin (acid) | CYP3A4 | 5.7775 | - | - | - | - |
| posaconazole | P-gp | 2 | - | - | - | - |
| posaconazole | CYP3A4 | 0.8575 | - | - | - | - |
| posaconazole | BCRP | 3 | - | - | - | - |
| quinidine | P-gp | 2.025 | - | - | - | - |
| quinidine | CYP3A4 | 14 | - | - | 0.65 | - |
| quinidine | CYP1A2 | 176.41 | - | - | - | - |
| quinidine | CES1 | 50.5 | - | - | - | - |
| rifampin | P-gp | 23 | - | - | - | - |
| rifampin | CYP3A4 | 18.5 | - | - | 8.25 | 0.4075 |
| rifampin | BCRP | 27.4 | - | - | - | - |
| rifampin | CYP1A2 | 3.11 | - | - | - | - |
| rifapentine | CYP3A4 | 11.4 | - | - | - | - |
| rifapentine | CYP1A2 | 123 | - | - | - | - |
| rifapentine | BCRP | 3.39 | - | - | - | - |
| ritonavir | CYP3A4 | 0.065 | 0.26 | 16.80 | 0.49 | - |
| ritonavir | P-gp | 2.20 | - | - | - | - |
| ritonavir | CYP2J2 | 3.395 | 0.51 | 2.52 | - | - |
| ritonavir | CYP1A2 | 68.75 | - | - | 1.2655 | - |
| ritonavir | BCRP | 9.39 | - | - | - | - |
| rosuvastatin (acid) | BCRP | 15.4 | - | - | - | - |
| rosuvastatin (acid) | CYP3A4 | 25.125 | - | - | - | - |
| ruxolitinib | P-gp | 11 | - | - | - | - |
| ruxolitinib | CYP3A4 | 4.4 | - | - | - | - |
| ruxolitinib | BCRP | 24 | - | - | - | - |
| sildenafil | CYP3A4 | 25.36 | - | - | - | - |
| sildenafil | P-gp | 8 | - | - | - | - |
| sildenafil | CYP1A2 | 150 | - | - | - | - |
| sildenafil | BCRP | 1.55 | - | - | - | - |
| sunitinib | P-gp | 7.6 | - | - | - | - |
| sunitinib | CYP3A4 | 36.675 | 16.73 | 1.71 | - | - |
| sunitinib | CYP1A2 | 5.4 | - | - | - | - |
| sunitinib | BCRP | 0.32 | - | - | - | - |
| sunitinib | CYP2J2 | 198.85 | - | - | - | - |
| tacrolimus | CYP3A4 | 0.36 | 2.66 | 18 | - | - |
| tacrolimus | P-gp | 0.42 | - | - | - | - |
| tacrolimus | CES2 | 9.29 | - | - | - | - |
| tacrolimus | BCRP | 3 | - | - | - | - |
| telaprevir | BCRP | 69.6 | - | - | - | - |
| telaprevir | P-gp | 2.05 | - | - | - | - |
| telaprevir | CYP3A4 | 5.55 | 1.01 | 3.90 | - | - |
| telaprevir | CES2 | 50 | - | - | - | - |
| telmisartan | CES2 | 1.2 | - | - | - | - |
| telmisartan | P-gp | 2.50 | - | - | - | - |
| telmisartan | CYP3A4 | 25 | - | - | - | - |
| telmisartan | CYP2J2 | 11.75 | - | - | - | - |
| telmisartan | CYP1A2 | 25 | - | - | - | - |
| telmisartan | BCRP | 0.31 | - | - | - | - |
| telmisartan | CES1 | - | - | - | - | - |
| teriflunomide | BCRP | 0.093 | - | - | - | - |
| teriflunomide | P-gp | 216 | - | - | - | - |
| teriflunomide | CYP3A4 | 130.75 | - | - | - | - |
| teriflunomide | CYP1A2 | 69 | - | - | - | - |
| ticagrelor | P-gp | 2.88 | - | - | - | - |
| ticagrelor | CYP3A4 | 4.1 | - | - | - | - |
| ticagrelor | BCRP | 0.18 | - | - | - | - |
| vandetanib | CYP2J2 | 5.46 | - | - | - | - |
| vandetanib | P-gp | 9.15 | - | - | - | - |
| vandetanib | BCRP | 10.75 | - | - | - | - |
| verapamil | P-gp | 8.11 | - | - | - | - |
| verapamil | CYP3A4 | 9.6 | 2.23 | 1.98 | 0.5 | - |
| verapamil | CYP2J2 | 12.2 | - | - | - | - |
| verapamil | CES2 | 9.795 | - | - | - | - |
| verapamil | CES1 | 16 | - | - | - | - |
| verapamil | CYP1A2 | 23 | - | - | - | - |
| verapamil | BCRP | 208.5 | - | - | - | - |
| ziprasidone | CYP3A4 | 36 | - | - | - | - |
| ziprasidone | CYP1A2 | 50 | - | - | - | - |
| ziprasidone | CES1 | 37.05 | - | - | - | - |
| ziprasidone | BCRP | 1.44 | - | - | - | - |
| *14-hydroxyclarithromycin* | CYP3A4 | 190 | - | - | - | - |
| *2-oxo-clopidogrel* | CYP3A4 | 4.1 | - | - | - | - |
| *2-oxo-clopidogrel* | CYP2J2 | - | - | - | - | - |
| *2-oxo-clopidogrel* | CYP1A2 | - | - | - | - | - |
| *2-oxo-clopidogrel* | CES1 | - | - | - | - | - |
| *clopidogrel acyl glucuronide* | CES1 | 24.80 | - | - | - | - |
| *clopidogrel acyl glucuronide* | CYP3A4 | 13.5 | - | - | - | - |
| *clopidogrel acyl glucuronide* | CYP2J2 | - | - | - | - | - |
| *clopidogrel acyl glucuronide* | CYP1A2 | - | - | - | - | - |
| *clopidogrel carboxylic acid* | CYP3A4 | 178 | - | - | - | - |
| *N,N-didesmethyldiltiazem* | CYP3A4 | 0.085 | - | - | - | - |
| *N-desmethyldiltiazem* | CYP3A4 | 0.8 | 0.77 | 1.62 | - | - |
| *N-desbutyldronedarone (SR35021)* | CYP3A4 | 32.715 | 6.24 | 5.94 | - | - |
| *N-desbutyldronedarone (SR35021)* | CYP2J2 | 1.54 | 0.48 | 1.5 | - | - |
| *N-desbutyldronedarone (SR35021)* | CYP1A2 | 30.6 | - | - | - | - |
| *N-desmethylimatinib (CGP74588)* | CYP3A4 | 13.7 | - | - | - | - |
| *N-desmethylimatinib (CGP74588)* | CYP1A2 | 32.50 | - | - | - | - |
| *N-desmethylimatinib (CGP74588)* | CYP2J2 | - | - | - | - | - |
| *N-desmethylimatinib (CGP74588)* | CES1 | - | - | - | - | - |
| *N-deacetylketoconazole* | CYP3A4 | 0.13 | - | - | - | - |
| *5-hydroxyomeprazole* | CYP3A4 | 10.5 | - | - | - | - |
| *5-hydroxyomeprazole* | CYP2J2 | - | - | - | - | - |
| *5-hydroxyomeprazole* | CYP1A2 | - | - | - | - | - |
| *5-hydroxyomeprazole* | CES1 | - | - | - | - | - |
| *ritonavir M1* | CYP3A4 | - | 1.4 | 19.8 | - | - |
| *telaprevir (R)-diastereomer VRT-127394* | CYP3A4 | 3.175 | - | - | - | - |
| *telaprevir (R)-diastereomer VRT-127394* | CYP2J2 | - | - | - | - | - |
| *telaprevir (R)-diastereomer VRT-127394* | CYP1A2 | - | - | - | - | - |
| *telaprevir (R)-diastereomer VRT-127394* | CES1 | - | - | - | - | - |
| *deshydroxyethoxy ticagrelor* | CYP3A4 | 0.75 | - | - | - | - |
| *deshydroxyethoxy ticagrelor* | CYP2J2 | - | - | - | - | - |
| *deshydroxyethoxy ticagrelor* | CYP1A2 | - | - | - | - | - |
| *deshydroxyethoxy ticagrelor* | CES1 | - | - | - | - | - |
| *N-dealkylverapamil* | CYP3A4 | - | 7.93 | 4.2 | - | - |
| *norverapamil* | CYP3A4 | 0.27 | 2.23 | 1.98 | - | - |
| *norverapamil* | CYP2J2 | 162 | - | - | - | - |

Italicized text denotes metabolites of the precipitants used in this study. The underlined values refer to half the IC_50_ as substitute to the reversible inhibition coefficient K_i_ in the case where the latter was not available in the DIDB. An enzyme induction scaling factor of d=1 was used in induction models in this study. Model K_deg_ values for CYP3A4 and CYP2J2 were taken from Fahmi et al. 2008, and Kosunen and Filppula 2023. -, not available.

# Supplemental Table 8. Single enzyme and transporter predictions as the effect of kinase inhibitors on the area under the concentration time curve of DOACs. For each DOAC, the maximum attainable inhibition for each mechanism is given for comparison.

| DOAC | Precipitant | CYP3A4_Total_ | CYP3A4_RI_ | CYP3A4_TDI_ | CYP1A2_RI_ | CYP2J2_RI_ | CES_RI_ | BCRP_RI_ | P-gp_RI_ | AUCR_Total_predicted_ |
| --- | --- | --- | --- | --- | --- | --- | --- | --- | --- | --- |
| apixaban | maximum inhibition | *1.25* | *1.25* | *1.25* | *1.08* | *1.08* |  | *1.15* | *1.26* | *2.33* |
| apixaban | afatinib | 1.00 | 1.00 |  |  |  |  | 1.15 | 1.22 | 1.46 |
| apixaban | axitinib | 1.09 | 1.00 | 1.09 | 1.01 |  |  | 1.11 | 1.20 | 1.51 |
| apixaban | erlotinib | 1.24 | 1.16 | 1.24 |  | 1.06 |  | 1.15 | 1.25 | 2.02 |
| apixaban | gefitinib | 1.23 | 1.04 | 1.22 |  | 1.04 |  | 1.15 | 1.25 | 1.96 |
| apixaban | imatinib | 1.24 | 1.10 | 1.23 | 1.00 | 1.05 |  | 1.15 | 1.24 | 2.00 |
| apixaban | lapatinib | 1.24 | 1.11 | 1.24 | 1.01 |  |  | 1.15 | 1.25 | 1.91 |
| apixaban | nilotinib | 1.24 | 1.23 | 1.24 | 1.01 | 1.08 |  | 1.15 | 1.25 | 2.11 |
| apixaban | pazopanib | 1.24 | 1.08 | 1.24 | 1.02 |  |  | 1.15 | 1.25 | 1.93 |
| apixaban | ruxolitinib | 1.01 | 1.01 |  |  |  |  | 1.09 | 1.19 | 1.35 |
| apixaban | sunitinib | 1.14 | 1.00 | 1.14 | 1.00 | 1.00 |  | 1.15 | 1.21 | 1.66 |
| apixaban | vandetanib |  |  |  |  | 1.05 |  | 1.15 | 1.25 | 1.58 |
| dabigatran etexilate | maximum inhibition |  |  |  |  |  |  |  | *2.57* | *2.99* |
| dabigatran etexilate | afatinib |  |  |  |  |  |  |  | 2.19 | 2.19 |
| dabigatran etexilate | axitinib |  |  |  |  |  |  |  | 2.01 | 2.01 |
| dabigatran etexilate | erlotinib |  |  |  |  |  |  |  | 2.45 | 2.45 |
| dabigatran etexilate | gefitinib |  |  |  |  |  |  |  | 2.52 | 2.52 |
| dabigatran etexilate | imatinib |  |  |  |  |  |  |  | 2.44 | 2.44 |
| dabigatran etexilate | lapatinib |  |  |  |  |  |  |  | 2.56 | 2.56 |
| dabigatran etexilate | nilotinib |  |  |  |  |  |  |  | 2.56 | 2.56 |
| dabigatran etexilate | pazopanib |  |  |  |  |  |  |  | 2.55 | 2.55 |
| dabigatran etexilate | ruxolitinib |  |  |  |  |  |  |  | 1.97 | 1.97 |
| dabigatran etexilate | sunitinib |  |  |  |  |  |  |  | 2.07 | 2.07 |
| dabigatran etexilate | vandetanib |  |  |  |  |  |  |  | 2.51 | 2.51 |
| edoxaban | maximum inhibition | *1.04* | *1.04* | *1.04* |  |  |  | *1.00* | *1.61* | *1.87* |
| edoxaban | afatinib | 1.00 | 1.00 |  |  |  |  | 1.00 | 1.50 | 1.51 |
| edoxaban | axitinib | 1.01 | 1.00 | 1.01 |  |  |  | 1.00 | 1.45 | 1.47 |
| edoxaban | erlotinib | 1.04 | 1.02 | 1.04 |  |  |  | 1.00 | 1.58 | 1.64 |
| edoxaban | gefitinib | 1.03 | 1.00 | 1.03 |  |  |  | 1.00 | 1.59 | 1.66 |
| edoxaban | imatinib | 1.04 | 1.01 | 1.04 |  |  |  | 1.00 | 1.57 | 1.64 |
| edoxaban | lapatinib | 1.04 | 1.02 | 1.04 |  |  |  | 1.00 | 1.60 | 1.67 |
| edoxaban | nilotinib | 1.04 | 1.03 | 1.04 |  |  |  | 1.00 | 1.60 | 1.67 |
| edoxaban | pazopanib | 1.04 | 1.01 | 1.04 |  |  |  | 1.00 | 1.60 | 1.67 |
| edoxaban | ruxolitinib | 1.00 | 1.00 |  |  |  |  | 1.00 | 1.43 | 1.44 |
| edoxaban | sunitinib | 1.02 | 1.00 | 1.02 |  |  |  | 1.00 | 1.47 | 1.51 |
| edoxaban | vandetanib |  |  |  |  |  |  | 1.00 | 1.59 | 1.59 |
| rivaroxaban | maximum inhibition | *1.21* | *1.21* | *1.21* |  | *1.16* |  | *1.15* | *1.26* | *2.22* |
| rivaroxaban | afatinib | 1.00 | 1.00 |  |  |  |  | 1.15 | 1.22 | 1.46 |
| rivaroxaban | axitinib | 1.08 | 1.00 | 1.08 |  |  |  | 1.11 | 1.20 | 1.48 |
| rivaroxaban | erlotinib | 1.21 | 1.14 | 1.21 |  | 1.12 |  | 1.15 | 1.25 | 2.11 |
| rivaroxaban | gefitinib | 1.20 | 1.03 | 1.20 |  | 1.08 |  | 1.15 | 1.25 | 2.00 |
| rivaroxaban | imatinib | 1.21 | 1.09 | 1.21 |  | 1.10 |  | 1.15 | 1.24 | 2.05 |
| rivaroxaban | lapatinib | 1.21 | 1.10 | 1.21 |  |  |  | 1.15 | 1.25 | 1.84 |
| rivaroxaban | nilotinib | 1.21 | 1.20 | 1.21 |  | 1.16 |  | 1.15 | 1.25 | 2.21 |
| rivaroxaban | pazopanib | 1.21 | 1.07 | 1.21 |  |  |  | 1.15 | 1.25 | 1.83 |
| rivaroxaban | ruxolitinib | 1.00 | 1.00 |  |  |  |  | 1.09 | 1.19 | 1.34 |
| rivaroxaban | sunitinib | 1.13 | 1.00 | 1.12 |  | 1.00 |  | 1.15 | 1.21 | 1.63 |
| rivaroxaban | vandetanib |  |  |  |  | 1.10 |  | 1.15 | 1.25 | 1.65 |

RI, reversible inhibition; TDI, time-dependent inhibition. Maximum inhibition is calculated as an f_m_- or f_e_-specific value.

# Supplemental Table 9*.* Summary of the drug Interaction clinical trial observations not included in model validation (*step 2*) and used to evaluate model predictions (*step 3*).

| Object |  | Precipitant | | | | |
| --- | --- | --- | --- | --- | --- | --- |
|  |  | atorvastatin (acid) | itraconazole | omeprazole | posaconazole | tacrolimus |
| apixaban | *Predicted* |  | 1.90 |  | 1.61 | 1.45 |
|  | *Observed* |  | 1.42^§^ |  | 1.62^§^ | 2.24 |
|  | *Predicted / observed ratio* |  | 1.33 |  | 0.99 | 0.64 |
| dabigatran etexilate | *Predicted* | 1.96 | 2.56 |  |  |  |
|  | *Observed* | No inhibition | 6.92^§^ |  |  |  |
|  | *Predicted / observed ratio* |  | 0.36 |  |  |  |
| edoxaban | *Predicted* | 1.46 | 1.67 |  | 1.62 |  |
|  | *Observed* | No inhibition | 1.86^§^ |  | 2.09^§^ |  |
|  | *Predicted / observed ratio* |  | 0.89 |  | 0.77 |  |
| rivaroxaban | *Predicted* | 1.50 | 1.84 | 1.54 | 1.60 |  |
|  | *Observed* | No inhibition | 1.47^§^ | No inhibition | 1.37^§^ |  |
|  | *Predicted / observed ratio* |  | 1.25 |  | 1.16 |  |

^§^ Trials which administered DOACs in microdoses. NOTE: The herein tabulated precipitants lacked C_max_ values which corresponded to the dosing used in their interaction trials. For this reason, they were not included in the model validation (*step 2*).

# Supplemental Table 10. Summary of the excluded microdose trials (step 2).

| Object | Precipitant | AUCR | Object Dose | Precipitant Dose | Precipitant Interval | Reference |
| --- | --- | --- | --- | --- | --- | --- |
| apixaban | cobicistat | 1.664 | 25 μg | 150 mg | 3 doses | Mikus et al., 2020 |
| apixaban | fluconazole | 1.098 | 25 μg | 400 mg | single dose | Rohr et al., 2022 |
| apixaban | isavuconazole | 1.326 | 25 μg | 200 mg (24 h) | TID | Rohr et al., 2022 |
| apixaban | itraconazole | 1.417 | 25 μg | 200 mg (24 h) | BID | Rohr et al., 2022 |
| apixaban | posaconazole | 1.618 | 25 μg | 300 mg (24 h) | BID | Rohr et al., 2022 |
| apixaban | ritonavir | 1.29 (1.04, 1.61) | 25 μg | 100 mg (5 days) | BID | Rohr et al., 2024 |
| apixaban | voriconazole | 1.327 | 25 μg | 200-400 mg | 6 doses | Mikus et al., 2020 |
| apixaban | voriconazole | 1.239 | 25 μg | 400 mg (24 h) | BID | Rohr et al., 2022 |
| dabigatran etexilate | itraconazole | 6.924 | 375 µg | 200 mg (5 days) | QD | Prueksaritanont et al., 2017 |
| edoxaban | clarithromycin | 2.03 (1.84, 2.24) | 50 μg | 500 mg | BID | Lenard et al., 2024 |
| edoxaban | cobicistat | 1.742 | 50 μg | 150 mg | 3 doses | Mikus et al., 2020 |
| edoxaban | fluconazole | 1.149 | 50 μg | 400 mg | single dose | Rohr et al., 2022 |
| edoxaban | isavuconazole | 1.515 | 50 μg | 200 mg (24 h) | TID | Rohr et al., 2022 |
| edoxaban | itraconazole | 1.857 | 50 μg | 200 mg (24 h) | BID | Rohr et al., 2022 |
| edoxaban | posaconazole | 2.089 | 50 μg | 300 mg (24 h) | BID | Rohr et al., 2022 |
| edoxaban | rifampin | 1.899 | 50 μg | 600 mg | single dose | Mikus et al., 2020 |
| edoxaban | ritonavir | 1.46 (1.23, 1.73) | 50 μg | 100 mg (5 days) | BID | Rohr et al., 2024 |
| edoxaban | voriconazole | 1.26 | 50 μg | 200-400 mg | 6 doses | Mikus et al., 2020 |
| edoxaban | voriconazole | 1.273 | 50 μg | 400 mg (24 h) | BID | Rohr et al., 2022 |
| rivaroxaban | cobicistat | 1.999 | 25 μg | 150 mg | 3 doses | Mikus et al., 2020 |
| rivaroxaban | isavuconazole | 1.133 | 25 μg | 200 mg (24 h) | TID | Rohr et al., 2022 |
| rivaroxaban | itraconazole | 1.472 | 25 μg | 200 mg (24 h) | BID | Rohr et al., 2022 |
| rivaroxaban | posaconazole | 1.372 | 25 μg | 300 mg (24 h) | BID | Rohr et al., 2022 |
| rivaroxaban | voriconazole | 1.312 | 25 μg | 200-400 mg | 6 doses | Mikus et al., 2020 |
| rivaroxaban | voriconazole | 1.165 | 25 μg | 400 mg (24 h) | BID | Rohr et al., 2022 |

All studies were of a cocktail study design in healthy volunteers. All drugs were orally administered. All object drugs were administered as single dose part of a cocktail solution.
